# Genetically Predicted Body Mass Index and Breast Cancer Risk: Mendelian Randomization Analyses of Data from 145,000 Women of European Descent

**DOI:** 10.1371/journal.pmed.1002105

**Published:** 2016-08-23

**Authors:** Yan Guo, Shaneda Warren Andersen, Xiao-Ou Shu, Kyriaki Michailidou, Manjeet K. Bolla, Qin Wang, Montserrat Garcia-Closas, Roger L. Milne, Marjanka K. Schmidt, Jenny Chang-Claude, Allison Dunning, Stig E. Bojesen, Habibul Ahsan, Kristiina Aittomäki, Irene L. Andrulis, Hoda Anton-Culver, Volker Arndt, Matthias W. Beckmann, Alicia Beeghly-Fadiel, Javier Benitez, Natalia V. Bogdanova, Bernardo Bonanni, Anne-Lise Børresen-Dale, Judith Brand, Hiltrud Brauch, Hermann Brenner, Thomas Brüning, Barbara Burwinkel, Graham Casey, Georgia Chenevix-Trench, Fergus J. Couch, Angela Cox, Simon S. Cross, Kamila Czene, Peter Devilee, Thilo Dörk, Martine Dumont, Peter A. Fasching, Jonine Figueroa, Dieter Flesch-Janys, Olivia Fletcher, Henrik Flyger, Florentia Fostira, Marilie Gammon, Graham G. Giles, Pascal Guénel, Christopher A. Haiman, Ute Hamann, Maartje J. Hooning, John L. Hopper, Anna Jakubowska, Farzana Jasmine, Mark Jenkins, Esther M. John, Nichola Johnson, Michael E. Jones, Maria Kabisch, Muhammad Kibriya, Julia A. Knight, Linetta B. Koppert, Veli-Matti Kosma, Vessela Kristensen, Loic Le Marchand, Eunjung Lee, Jingmei Li, Annika Lindblom, Robert Luben, Jan Lubinski, Kathi E. Malone, Arto Mannermaa, Sara Margolin, Frederik Marme, Catriona McLean, Hanne Meijers-Heijboer, Alfons Meindl, Susan L. Neuhausen, Heli Nevanlinna, Patrick Neven, Janet E. Olson, Jose I. A. Perez, Barbara Perkins, Paolo Peterlongo, Kelly-Anne Phillips, Katri Pylkäs, Anja Rudolph, Regina Santella, Elinor J. Sawyer, Rita K. Schmutzler, Caroline Seynaeve, Mitul Shah, Martha J. Shrubsole, Melissa C. Southey, Anthony J. Swerdlow, Amanda E. Toland, Ian Tomlinson, Diana Torres, Thérèse Truong, Giske Ursin, Rob B. Van Der Luijt, Senno Verhoef, Alice S. Whittemore, Robert Winqvist, Hui Zhao, Shilin Zhao, Per Hall, Jacques Simard, Peter Kraft, Paul Pharoah, David Hunter, Douglas F. Easton, Wei Zheng

**Affiliations:** 1 Department of Cancer Biology, Vanderbilt University, Nashville, Tennessee, United States of America; 2 Division of Epidemiology, Department of Medicine, Vanderbilt-Ingram Cancer Center, Vanderbilt University School of Medicine, Nashville, Tennessee, United States of America; 3 Centre for Cancer Genetic Epidemiology, Department of Public Health and Primary Care, University of Cambridge, Cambridge, United Kingdom; 4 Division of Genetics and Epidemiology, The Institute of Cancer Research, London, United Kingdom; 5 Breakthrough Breast Cancer Research Centre, The Institute of Cancer Research, London, United Kingdom; 6 Cancer Epidemiology Centre, Cancer Council Victoria, Melbourne, Australia; 7 Centre for Epidemiology and Biostatistics, School of Population and Global health, The University of Melbourne, Melbourne, Australia; 8 Netherlands Cancer Institute, Antoni van Leeuwenhoek hospital, Amsterdam, The Netherlands; 9 Division of Cancer Epidemiology, German Cancer Research Center (DKFZ), Heidelberg, Germany; 10 Department of Obstetrics and Gynecology, University of Ulm, Ulm, Germany; 11 Academic Unit of Pathology, Department of Neuroscience, University of Sheffield, Sheffield, United Kingdom; 12 Faculty of Health and Medical Sciences, University of Copenhagen, Copenhagen, Denmark; 13 Department of Clinical Biochemistry, Herlev Hospital, Copenhagen University Hospital, Herlev, Denmark; 14 Copenhagen General Population Study, Herlev Hospital, Copenhagen University Hospital, Herlev, Denmark; 15 Department of Health Studies, The University of Chicago, Chicago, Illinois, United States of America; 16 Department of Clinical Genetics, Helsinki University Hospital, University of Helsinki, Helsinki, Finland; 17 Lunenfeld-Tanenbaum Research Institute of Mount Sinai Hospital, Toronto, Canada; 18 Department of Molecular Genetics, University of Toronto, Toronto, Canada; 19 Department of Epidemiology, University of California Irvine, Irvine, California, United States of America; 20 Division of Clinical Epidemiology and Aging Research, German Cancer Research Center (DKFZ), Heidelberg, Germany; 21 Human Cancer Genetics Program, Spanish National Cancer Research Centre, Madrid, Spain; 22 Centro de Investigación en Red de Enfermedades Raras, Valencia, Spain; 23 Department of Radiation Oncology, Hannover Medical School, Hannover, Germany; 24 Division of Cancer Prevention and Genetics, Istituto Europeo di Oncologia, Milan, Italy; 25 Department of Genetics, Institute for Cancer Research, Radiumhospitalet, Oslo University Hospital, Oslo University Hospital, Oslo, Norway; 26 Institute of Clinical Medicine, Faculty of Medicine, University of Oslo, Oslo, Norway; 27 Department of Medical Epidemiology and Biostatistics, Karolinska Institutet, Stockholm, Sweden; 28 German Cancer Consortium (DKTK), German Cancer Research Center (DKFZ), Heidelberg, Germany; 29 Dr. Margarete Fischer-Bosch-Institute of Clinical Pharmacology, Stuttgart, Germany; 30 University of Tübingen, Tübingen, Germany; 31 Division of Preventive Oncology, German Cancer Research Center (DKFZ), Heidelberg, Germany; 32 Institute for Prevention and Occupational Medicine of the German Social Accident Insurance, Institute of the Ruhr University Bochum, Bochum, Germany; 33 Division of Molecular Genetic Epidemiology, German Cancer Research Center (DKFZ), Heidelberg, Germany; 34 Molecular Epidemiology Group, German Cancer Research Center (DKFZ), Heidelberg, Germany; 35 Department of Preventive Medicine, Keck School of Medicine, University of Southern California, Los Angeles, California, United States of America; 36 Department of Genetics, QIMR Berghofer Medical Research Institute, Brisbane, Australia; 37 Department of Laboratory Medicine and Pathology, Mayo Clinic, Rochester, Minnesota, United States of America; 38 Sheffield Cancer Research, Department of Oncology, University of Sheffield, Sheffield, United Kingdom; 39 Department of Human Genetics, Leiden University Medical Center, Leiden, The Netherlands; 40 Gynaecology Research Unit, Hannover Medical School, Hannover, Germany; 41 Centre Hospitalier Universitaire de Québec Research Center, Laval University, Québec City, Canada; 42 Department of Gynaecology and Obstetrics, University Hospital Erlangen, Friedrich-Alexander University Erlangen-Nuremberg, Comprehensive Cancer Center Erlangen-EMN, Erlangen, Germany; 43 David Geffen School of Medicine, Department of Medicine Division of Hematology and Oncology, University of California at Los Angeles, Los Angeles, California, United States of America; 44 Division of Cancer Epidemiology and Genetics, National Cancer Institute, Rockville, Maryland, United States of America; 45 Institute for Medical Biometrics and Epidemiology, University Medical Center Hamburg-Eppendorf, Hamburg, Germany; 46 Department of Cancer Epidemiology, Clinical Cancer Registry, University Medical Center Hamburg-Eppendorf, Hamburg, Germany; 47 Department of Breast Surgery, Herlev Hospital, Copenhagen University Hospital, Herlev, Denmark; 48 Molecular Diagnostics Laboratory, IRRP, National Centre for Scientific Research "Demokritos", Athens, Greece; 49 Departments of Epidemiology, University of North Carolina Chapel-Hill, Chapel Hill, North Carolina, United States of America; 50 Environmental Epidemiology of Cancer, Center for Research in Epidemiology and Population Health, INSERM, Villejuif, France; 51 University Paris-Sud, Villejuif, France; 52 Molecular Genetics of Breast Cancer, German Cancer Research Center (DKFZ), Heidelberg, Germany; 53 Department of Surgical Oncology, Erasmus University Medical Center, Rotterdam, The Netherlands; 54 Department of Genetics and Pathology, Pomeranian Medical University, Szczecin, Poland; 55 Department of Epidemiology, Cancer Prevention Institute of California, Fremont, California, United States of America; 56 Department of Health Research and Policy, Stanford University School of Medicine, Stanford, California, United States of America; 57 Prosserman Centre for Health Research, Lunenfeld-Tanenbaum Research Institute of Mount Sinai Hospital, Toronto, Canada; 58 Division of Epidemiology, Dalla Lana School of Public Health, University of Toronto, Toronto, Canada; 59 Imaging Center, Department of Clinical Pathology, Kuopio University Hospital, Kuopio, Finland; 60 Institute of Clinical Medicine, Pathology and Forensic Medicine, University of Eastern Finland, Kuopio, Finland; 61 Cancer Center of Eastern Finland, University of Eastern Finland, Kuopio, Finland; 62 Department of Clinical Molecular Biology, Oslo University Hospital, University of Oslo, Oslo, Norway; 63 University of Hawaii Cancer Center, Honolulu, Hawaii, United States of America; 64 Department of Molecular Medicine and Surgery, Karolinska Institutet, Stockholm, Sweden; 65 Clinical Gerontology, Department of Public Health and Primary Care, University of Cambridge, Cambridge, United Kingdom; 66 Division of Public Health Sciences, Fred Hutchinson Cancer Research Center, Seattle, Washington, United States of America; 67 Department of Oncology – Pathology, Karolinska Institutet, Stockholm, Sweden; 68 National Center for Tumor Diseases, University of Heidelberg, Heidelberg, Germany; 69 Department of Obstetrics and Gynecology, University of Heidelberg, Heidelberg, Germany; 70 Anatomical Pathology, The Alfred Hospital, Melbourne, Australia; 71 Department of Clinical Genetics, VU University Medical Center, Amsterdam, The Netherlands; 72 Division of Gynaecology and Obstetrics, Technische Universität München, Munich, Germany; 73 Beckman Research Institute of City of Hope, Duarte, California, United States of America; 74 Department of Obstetrics and Gynecology, Helsinki University Hospital, University of Helsinki, Helsinki, Finland; 75 Department of Oncology, University Hospital Gasthuisberg, Leuven, Belgium; 76 Department of Health Sciences Research, Mayo Clinic, Rochester, Minnesota, United States of America; 77 Servicio de Cirugía General y Especialidades, Hospital Monte Naranco, Oviedo, Spain; 78 Centre for Cancer Genetic Epidemiology, Department of Oncology, University of Cambridge, Cambridge, United Kingdom; 79 IFOM, Fondazione Istituto FIRC (Italian Foundation of Cancer Research) di Oncologia Molecolare, Milan, Italy; 80 Peter MacCallum Cancer Center, The University of Melbourne, Melbourne, Australia; 81 Sir Peter MacCallum Department of Oncology, The University of Melbourne, Melbourne, Australia; 82 Department of Medicine, St Vincent’s Hospital, The University of Melbourne, Fitzroy, Australia; 83 Laboratory of Cancer Genetics and Tumor Biology, Department of Clinical Chemistry and Biocenter Oulu, University of Oulu, Oulu, Finland; 84 Herbert Irving Comprehensive Cancer Center, Columbia University Medical Center, New York, New York, United States of America; 85 Department of Environmental Health Sciences, Mailman School of Public Health of Columbia University, New York, New York, United States of America; 86 Research Oncology, Guy’s Hospital, King's College London, London, United Kingdom; 87 Division of Molecular Gyneco-Oncology, Department of Gynaecology and Obstetrics, University Hospital of Cologne, Cologne, Germany; 88 Center for Integrated Oncology, University Hospital of Cologne, Cologne, Germany; 89 Center for Molecular Medicine, University Hospital of Cologne, Cologne, Germany; 90 Center of Familial Breast and Ovarian Cancer, University Hospital of Cologne, Cologne, Germany; 91 Department of Pathology, The University of Melbourne, Melbourne, Australia; 92 Division of Breast Cancer Research, The Institute of Cancer Research, London, United Kingdom; 93 Department of Molecular Virology, Immunology and Medical Genetics, Comprehensive Cancer Center, The Ohio State University, Columbus, Ohio, United States of America; 94 Wellcome Trust Centre for Human Genetics and Oxford Biomedical Research Centre, University of Oxford, Oxford, United Kingdom; 95 Department of Nutrition, Institute of Basic Medical Sciences, University of Oslo, Oslo, Norway; 96 Department of Medical Genetics, University Medical Center Utrecht, Utrecht, The Netherlands; 97 Laboratory of Cancer Genetics and Tumor Biology, Northern Finland Laboratory Centre NordLab, Oulu, Finland; 98 Vesalius Research Center, Leuven, Belgium; 99 Laboratory for Translational Genetics, Department of Oncology, University of Leuven, Leuven, Belgium; 100 Program in Genetic Epidemiology and Statistical Genetics, Harvard School of Public Health, Boston, Massachusetts, United States of America; 101 Department of Epidemiology, Harvard School of Public Health, Boston, Massachusetts, United States of America; Harvard Medical School, UNITED STATES

## Abstract

**Background:**

Observational epidemiological studies have shown that high body mass index (BMI) is associated with a reduced risk of breast cancer in premenopausal women but an increased risk in postmenopausal women. It is unclear whether this association is mediated through shared genetic or environmental factors.

**Methods:**

We applied Mendelian randomization to evaluate the association between BMI and risk of breast cancer occurrence using data from two large breast cancer consortia. We created a weighted BMI genetic score comprising 84 BMI-associated genetic variants to predicted BMI. We evaluated genetically predicted BMI in association with breast cancer risk using individual-level data from the Breast Cancer Association Consortium (BCAC) (cases  =  46,325, controls  =  42,482). We further evaluated the association between genetically predicted BMI and breast cancer risk using summary statistics from 16,003 cases and 41,335 controls from the Discovery, Biology, and Risk of Inherited Variants in Breast Cancer (DRIVE) Project. Because most studies measured BMI after cancer diagnosis, we could not conduct a parallel analysis to adequately evaluate the association of measured BMI with breast cancer risk prospectively.

**Results:**

In the BCAC data, genetically predicted BMI was found to be inversely associated with breast cancer risk (odds ratio [OR]  =  0.65 per 5 kg/m^2^ increase, 95% confidence interval [CI]: 0.56–0.75, *p* = 3.32 × 10^−10^). The associations were similar for both premenopausal (OR   =   0.44, 95% CI:0.31–0.62, *p*  =  9.91 × 10^−8^) and postmenopausal breast cancer (OR  =  0.57, 95% CI: 0.46–0.71, *p*  =  1.88 × 10^−8^). This association was replicated in the data from the DRIVE consortium (OR  =  0.72, 95% CI: 0.60–0.84, *p*   =   1.64 × 10^−7^). Single marker analyses identified 17 of the 84 BMI-associated single nucleotide polymorphisms (SNPs) in association with breast cancer risk at *p* < 0.05; for 16 of them, the allele associated with elevated BMI was associated with reduced breast cancer risk.

**Conclusions:**

BMI predicted by genome-wide association studies (GWAS)-identified variants is inversely associated with the risk of both pre- and postmenopausal breast cancer. The reduced risk of postmenopausal breast cancer associated with genetically predicted BMI observed in this study differs from the positive association reported from studies using measured adult BMI. Understanding the reasons for this discrepancy may reveal insights into the complex relationship of genetic determinants of body weight in the etiology of breast cancer.

## Abstract 2

Using Mendelian randomization analysis, Wei Zheng and colleagues probe potentially causal associations between BMI and breast cancer risk in both pre- and postmenopausal women.

## Author Summary

**Why Was This Study Done?:**

- Body mass index (BMI) has been linked to breast cancer risk in conventional population studies.
- In these studies, high BMI is associated with reduced risk of breast cancer in premenopausal women but with increased risk in postmenopausal women. These changed risks may be caused by BMI or caused by environmental factors that are associated with BMI.
- We sought to use a research tool from the genetics field to understand BMI’s causal role in breast cancer.

**What Did the Researchers Do and Find?:**

- We took advantage of previously identified genetic sequence variations that are associated with BMI in European populations and used these variants to predict BMI. These variants are set at birth and are not affected by environmental factors; thus, outcomes associated with high BMI as predicted by genetic variants are more likely to be caused by high BMI itself rather than by environmental factors that are associated with high BMI.
- Using databases containing individual genetic sequences and breast cancer diagnoses in a European population, we tested whether genetically predicted BMI was associated with diagnosis of breast cancer in either pre- or postmenopausal women.
- We found that genetically predicted high BMI was associated with decreased breast cancer risk, in both cancer databases. Unexpectedly, this was true for both pre- and postmenopausal women.

**What Do These Findings Mean?:**

- Our results from postmenopausal women contradict prior findings from population studies, which used measured, rather than genetically predicted, BMI.
- BMI predicted using genetic variants identified to date may be more closely related to body weight in early life or midlife, which is negatively associated with risk of breast cancer. Measured high BMI later in life may be influenced by environmental factors that are associated with increased risk of breast cancer.
- More research is needed on the interrelationship of genetic factors, environment, and BMI in the risk of breast cancer.

## Introduction

The association between body mass index (BMI) and breast cancer risk has been extensively investigated in observational epidemiologic studies. Most prospective cohort studies reported an inverse association between BMI and premenopausal breast cancer risk [1–7]. A modest positive association has been reported between BMI and postmenopausal breast cancer risk [1,3,8], and this association was primarily limited to women who did not use postmenopausal hormone therapy (HT) [2,9,10] or women diagnosed with estrogen receptor (ER)-positive breast cancer [10].

Several explanations have been proposed for the opposite direction of the association between BMI and breast cancer risk by menopausal status. For example, it is postulated that overweight and obese women are more likely to experience anovulatory menstrual cycles, potentially leading to lower exposure to ovarian hormones and thus reducing the risk of breast cancer in premenopausal women [11,12]. Among postmenopausal women, the primary source of estrogen is the conversion of androgens in adipose tissue. Overweight women have been found to have higher estrogen levels than normal weight women, providing a possible explanation for positive associations observed between BMI and breast cancer risk in postmenopausal women. Although these postulated explanations are biologically plausible for the different associations observed between measured BMI and breast cancer risk in pre-and postmenopausal women, it remains unclear whether BMI is causally associated with breast cancer risk or serves as a surrogate measure for other risk factors. These uncertainties should be clearly communicated in public health messages about breast cancer prevention.

Recent genome-wide association studies (GWAS) have identified multiple loci associated with BMI. A genetic score, comprising BMI-associated single nucleotide polymorphisms (SNPs) capturing the portion of BMI determined by genetic factors, can be used in Mendelian randomization (MR) as the instrumental variable to evaluate the association between BMI and breast cancer risk by eliminating concerns of reverse causation and reducing the likelihood of selection bias and confounding in conventional epidemiologic studies. This is because the alleles associated with BMI should be randomly assigned to offspring from parents during gamete formation. In this study, data from two large consortia were used to conduct a MR analysis to assess the association between BMI and breast cancer risk.

## Methods

### Study Population: BCAC and DRIVE Consortia

We obtained data from two large consortia, the Breast Cancer Association Consortium (BCAC) and the Discovery, Biology, and Risk of Inherited Variants in Breast Cancer (DRIVE) Project. All participating studies obtained written, informed consent from all subjects and received study protocol approval from their respective Institutional Review Boards. Our first analysis included 39 studies contributing participants of European ancestry to the BCAC Collaborative Oncological Gene Environment Study (COGS) project (S1 Table). This analysis included data from 46,235 breast cancer cases and 42,482 controls. Selected characteristics of BCAC participants by study are provided in S2 Table. Details of the genotyping protocol in the BCAC are described elsewhere [13] (http://ccge.medschl.cam.ac.uk/research/consortia/icogs/). Genotype data were obtained either by direct genotyping using a custom Illumina iSelect genotyping array (iCOGS) that contains 211,155 SNPs [13] or by imputation, using data from the iCOGS array and the 1000 Genomes Project Phase I integrated variant set (March 2012 release) as the reference using the program IMPUTE2 [14]. Population-specific variations in allele frequencies of the SNPs were accounted for by eight principal components using a set of 37,000 uncorrelated SNPs, including those selected as ancestry-informative markers, as previously described [13].

To further assess the association between genetically predicted BMI and breast cancer risk, we analyzed data from the DRIVE project, for which summary-level statistics from 16,003 breast cancer cases and 41,335 controls of European ancestry from 11 participating studies were available (S3 Table). DRIVE project genotyping data were generated by Illumina and Affymetrix SNP genotyping arrays or by genotype imputation with the HapMap phase 2 CEU panel as reference using MACH v1.0 [15] or IMPUTE [14].

### Selection of BMI-Associated SNPs

SNPs associated with variation in BMI were identified from the NHGRI-EBI Catalog of Published Genome-Wide Association Studies in August 2015 [16]. Furthermore, we included all BMI-associated SNPs from the latest finding of Genetic Investigation of Anthropometric Traits (GIANT) [17]. SNPs associated with BMI at genome-wide significance levels (*p* < 5 × 10^−8^) in populations of European ancestry were selected for this study. We selected independent SNPs, defined as r^2^ < 0.1 based on International HapMap Project phase 3 data. For any two SNPs with an r^2^ ≥ 0.1, the SNP with the lower *p*-value for association with BMI was selected. In total, 84 SNPs were selected for analysis. In BCAC data, 50 of the 84 SNPs were successfully genotyped, and the remaining 34 SNPs were imputed with high quality (imputation *r* ^2^ > 0.8).

### Statistical Analysis

Genetic scores for BMI (BMI-GS) used for MR were computed using previously described methods [18–22]. The GS used in our primary analysis was constructed using external weights, and calculated using the following formula: $GS=\sum_{i=1}^{84}\beta_{i}SNP_{i}$, where *β*_*i*_ is the effect of the *i*th SNP for BMI reported in previous studies [17] and *SNP*_*i*_ is the dosage of the effect allele (range: 0 to 2) of the *i*th SNP. To scale the GS to the unit of BMI, we first performed a linear regression among controls, observed BMI ~ GS + error, where the expectation of error is zero. From this regression we obtained *β*_*0*_ (slope = 18.99) and *β*_*1*_ (effect = 0.451). Then, we used the values of *β*_*0*_ and *β*_*1*_ to compute BMI-GS using the formula, BMI-GS = *β*_*0*_ *+ β*_*1*_ * GS. The BMI-GS is a linear transformation of GS, and thus, these two variables were perfectly correlated (r = 1.0).

Pooled analyses and meta-analysis were conducted to evaluate the association of BMI-GS with breast cancer risk. In pooled analysis, subjects from all BCAC studies were analyzed with adjustment for the BCAC study sites. In meta-analysis, we estimated the risk of breast cancer associated with BMI-GS in each of the BCAC studies, and then combined the results using a fixed effects model. Sensitivity analyses were performed using an unweighted BMI-GS to evaluate the robustness of the association (S4 Table). The percentage of BMI variation explained by BMI-GS was calculated using linear regression models. We performed Egger regression [23] analysis to detect possible pleiotropic effects of the instrumental variable used in our analyses.

Logistic regression was used to calculate adjusted odds ratios for the association between BMI-GS (continuously and categorically: 25.5–25.9, 26.0–26.5, and ≥26.5 kg/m^2^), versus <25.5. Traditional World Health Organization BMI cutoffs were not used because of the narrow range of the BMI predicted by BMI-GSs (range: 24.14–28.53).

We performed stratified analyses by factors that could potentially modify the association, including age, menopausal status, and postmenopausal HT. We assessed heterogeneity by hormone receptor status. Potential confounders included in logistic regression models were BCAC study site, age, and the eight principal components as described previously [13]. In some analyses, we also adjusted for known and suspected breast cancer risk factors, including age at menarche, HT use, and smoking. We used the two-sample method [24] to analyze the association of BMI-GS and breast cancer risk using the summary statistics data obtained from the DRIVE project (available on the Genetic Associations and Mechanisms in Oncology [GAME-ON] website: http://gameon.dfci.harvard.edu). The potential causal association between BMI (*X*) and breast cancer risk (*Y*) was modeled using BMI-associated SNPs as the instrumental variable [25]. Specifically, the causal effect (*β*_*YX*_) was calculated by using the Wald estimator: $\beta_{YX}=\frac{\beta_{YG}}{\beta_{XG}}$, where *β*_*YG*_ is the natural log-scale odds ratio (OR) for breast cancer risk associated with the instrumental variable; *β*_*XG*_ is the regression coefficient of the instrumental variable for BMI obtained from previous GWAS [17]. The standard error for the causal effect was computed using the delta method [26]: $SE_{YX}=\sqrt{\left({\left(\frac{S_{YG}}{\beta_{XG}}\right)}^{2}+\frac{{\left(S_{XG}\beta_{YG}\right)}^{2}}{\beta_{XG}{}^{4}}\right)}$; *S*_*YG*_ and *S*_*XG*_ are the corresponding standard errors. We used an inverse-variance weighted method [27] to evaluate the combined association of the 84 BMI-associated SNPs with breast cancer risk.

To evaluate the associations between individual SNPs and breast cancer risk, summary estimates from the BCAC and DRIVE datasets were combined using the inverse-variance weighted method [28]. Analyses were performed using PLINK (v 1.07), R (v 3.02), and SAS (v 9.3). A two-sided *p*-value < 0.05 was considered statistically significant unless stated otherwise.

## Results

In pooled analyses including BCAC controls, the point estimates for the associations between all 84 SNPs and BMI were in the same direction as reported in the literature. However, only 39 of the 84 SNPs showed associations with BMI at *p ≤ *0.05, likely because of small sample size (S5 Table).

As expected, we observed a positive association between BMI-GS and observed BMI in pooled analyses using data from controls (*p* < 0.001 for premenopausal women, *p* < 0.001for postmenopausal women, and *p* < 0.001for all controls combined) (Table 1). Using data from cases and controls combined, we showed associations of BMI-GS with age at menarche (*p* < 0.001), postmenopausal HT use (*p*  = 0.004), smoking (*p *< 0.001), and weight (*p*  < 0.001). Results were unchanged after adjusting for observed BMI (S6 Table).

**Table 1:** Associations of the weighted BMI-GSs with BMI and traditional breast cancer risk factors.

Outcome	Number of Participants	Summary Effect*	Standard Error	*P*-value
**BMI (kg/m** ^**2**^ **)** †				
Controls	22,056	0.451	0.0286	1.55 × 10^−55^
Premenopausal controls	5,532	0.456	0.0565	9.38 × 10^−16^
Postmenopausal controls	15,025	0.449	0.0345	4.96 × 10^−38^
**Traditional Risk Factors** **				
Age (years)	88,807	0.0012	0.0034	0.71
Age at menarche (years)	53,990	−0.0719	0.0061	4.06 × 10^−32^
Menopausal status (post versus pre)	61,686	0.0044	0.0082	0.59
Age at menopause (years)	26,921	0.0359	0.0322	0.26
Family history of breast cancer (yes versus no)	47,417	−0.0102	0.0111	0.36
Parous (yes versus no)	62,683	0.0118	0.0103	0.25
Parity (numbers)	61,837	0.0049	0.0049	0.32
Age at first live birth (years)	44,735	−0.0563	0.0206	0.006
Use of HRT (postmenopausal) (ever versus never)	22,400	−0.0367	0.0128	0.004
Breastfeeding (ever versus never)	43,321	0.0125	0.0095	0.19
Smoking (ever versus never)	39,562	0.0305	0.009	0.0007
Weight (control) (kg)	15,410	1.3769	0.0971	2.35 × 10^−45^
Height (cm)	50,706	0.0336	0.0255	0.19

HRT, hormone replacement therapy. The results stratified by menopausal status for significant risk factors are as follows: formatted as (summary effect, standard error, and *p*-value); age at menarche: premenopausal (−0.0802, 0.0106, 6.63 × 10^−14^) and postmenopausal (−0.0099, 0.001, 5.75 × 10^−23^); age at first birth: premenopausal (−0.0634, 0.0392, 0.11) and postmenopausal (−0.0431, 0.0246, 0.08); smoking: premenopausal (0.0382, 0.0168, 0.02) and postmenopausal (0.0285, 0.0109, 0.009); and weight: premenopausal (1.4767, 0.2133, 5.31 × 10^−12^) and postmenopausal: (1.3893, 0.1175, 5.24 × 10^−32^).

* The regression coefficient is presented for continuous variables and natural log-scale OR for dichotomous variables, per unit increase of the weighted BMI-GS.

^†^ There was no heterogeneity in the association of the weighted BMI-GS with observed BMI among cases and controls.

** The linear regression models fitting weight included only controls; models of all other traditional breast cancer risk factors included all subjects. The total number of subjects is 88,807 (cases + controls) in our dataset. A total of 22,056 controls have observed BMI. The premenopausal controls and the postmenopausal controls do not add up to the total number of controls because of missing menopausal status.

**Fig 1:**
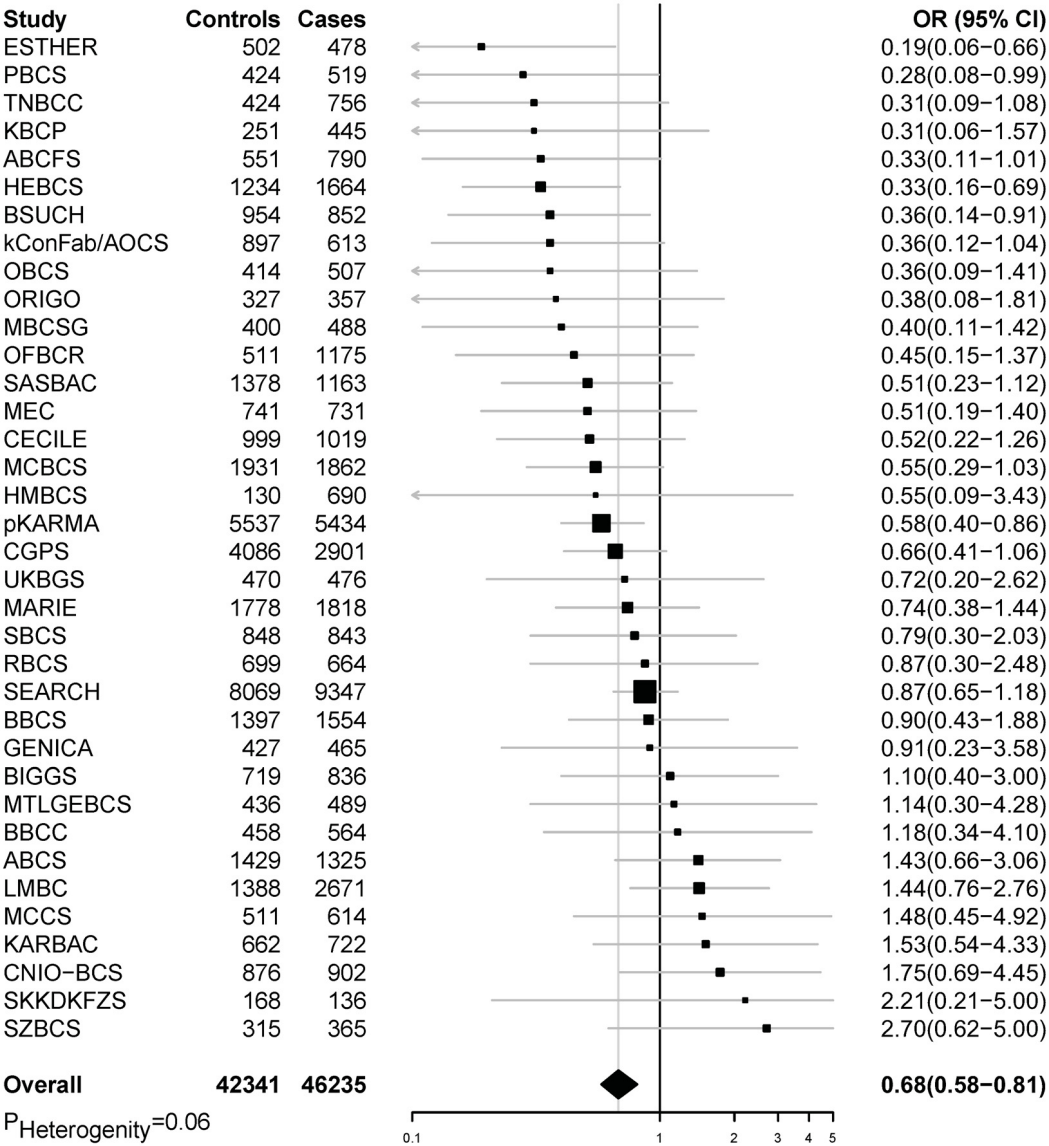
Meta-analysis of the association between genetically predicted BMI and breast cancer risk in the BCAC. The summary OR was calculated by combining individual analysis results from each study in BCAC (*p* for heterogeneity = 0.06).

**Fig 2:**
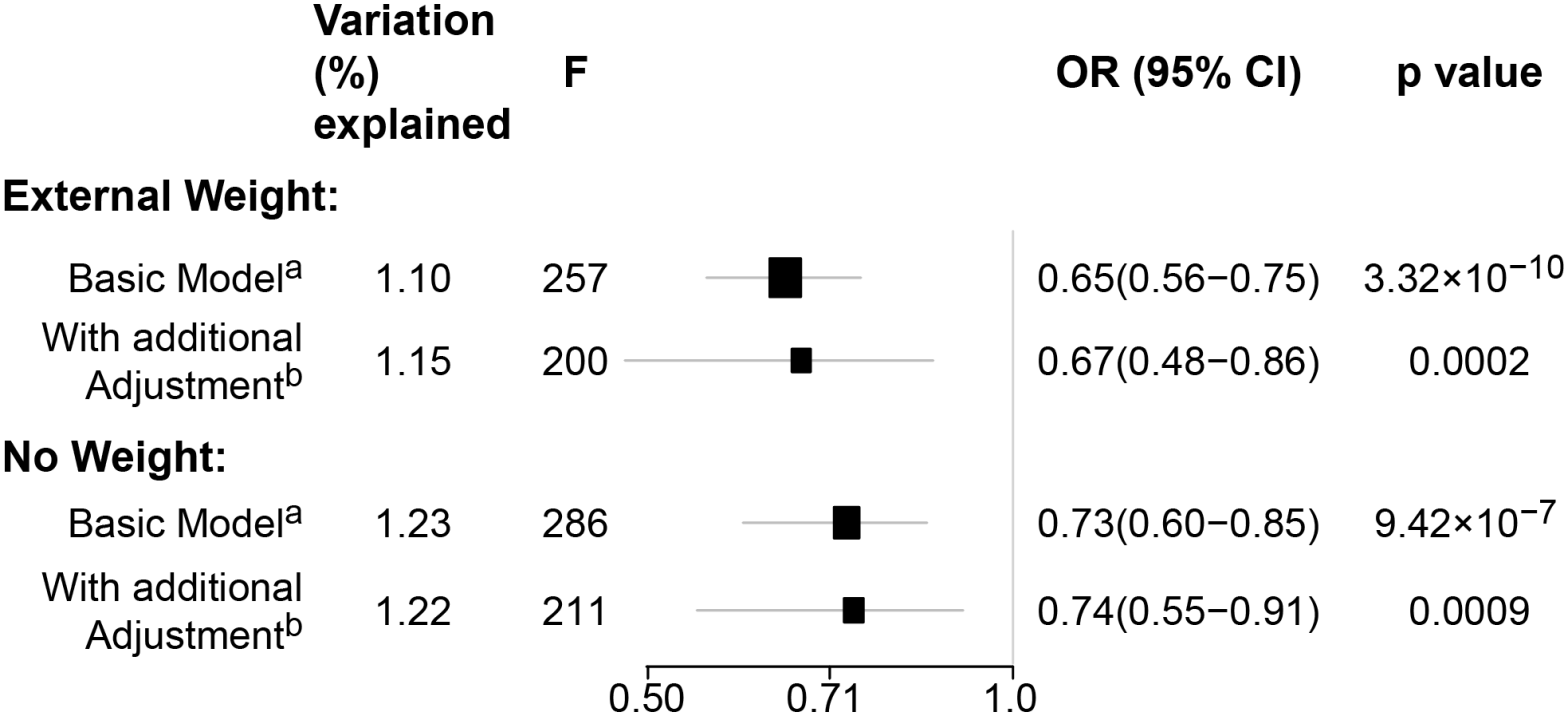
Sensitivity analyses using pooled data for associations between genetically predicted BMI and breast cancer risk in the BCAC. (A) Adjusted for age, study sites, and the first eight principal components. (b) Adjusted for age, study sites, the first eight principal components, and additional breast cancer risk factors: age at menarche, parity, use of contraceptive, use of hormone replacement therapy, breast feeding, and smoking status. Weighted: the BMI-GS was constructed using the additive model weighted by external beta reported from previous literatures. Unweighted: the BMI-GS was constructed using the additive model without any weight.

In pooled analyses of BCAC data, an inverse association was observed between breast cancer risk and genetically predicted BMI (Table 2). The OR per 5 kg/m^2^ increase in BMI using meta-analyses was 0.65 (95% CI: 0.56–0.75, *p* < 3.32×10^−10^), which was similar to that derived from the pooled analyses, OR = 0.68 (95% CI: 0.58–0.81, *p* < 2.50 × 10^−5^). There was no apparent evidence for heterogeneity in the OR among BCAC studies (heterogeneity *p* = 0.06) (Fig 1). MR-Egger regression testing on funnel plot asymmetry yielded *p* = 0.44, suggesting no violation of the basic assumptions for MR (S1 Fig). In pooled analysis, adjusting for observed BMI did not change the results (OR = 0.57, 95% CI: 0.45–0.70, *p*  = 8.17×10^−9^ *)*. As a part of the sensitivity analysis, we also performed pooled analysis adjusting for breast cancer risk factors as covariables. As expected, adjustment of these variables slightly attenuated the association. However, the association remained highly statistically significant (Fig 2). The OR for the association between genetically predicted BMI and breast cancer risk was similar for pre- and postmenopausal women (heterogeneity test, *p* = 0.45), and in postmenopausal women, it was similar for women with and without use of HT (heterogeneity test, *p* = 0.42). There was some evidence for a stronger association for ER-positive tumors than ER-negative tumors (heterogeneity test, ER *p* = 0.03). Associations were similar in population-based studies (OR = 0.52, 95% CI: 0.38–0.70, *p*  = 1.84×10^−6^) and non-population-based studies (OR = 0.71, 95% CI: 0.54–0.92, *p*  = 0.007). Analyses using categorical variables of genetically predicted BMI showed inverse results similar to analyses treating predicted BMI as a continuous variable. We also stratified subjects by age (<50 y, 50–55 y, 55–65 y, >65 y) and found an inverse association between genetically predicted BMI and breast cancer risk for all age groups ≤ 65 y (S7 Table). No association between predicted BMI and breast cancer was observed in the age group > 65 (OR = 0.85, 95% CI: 0.62–1.15, *p* = 0.29). The BMI predicted using unweighted GS was also associated with reduced breast cancer risk (Fig 2). The effect sizes were similar, but somewhat weaker for unweighted analyses. The BMI-GS explained 1.23% of variation in BMI in the BCAC control group. Analyses of summary statistics from the DRIVE project replicated the inverse association between genetically predicted BMI and breast cancer risk, OR = 0.72 (95% CI: 0.60–0.84, *p* = 1.64×10^−7^) (S8 Table). The strength of the association observed was similar to that observed in the BCAC dataset.

**Table 2:** Associations between genetically predicted BMI and breast cancer risk.

	By BMI Group*				Per 5 kg/m^2^ Increase †	
	Subjects	25.5–25.9	26.0–26.5	≥26.5		
		OR (95% CI)	OR (95% CI)	OR (95% CI)	OR (95% CI)	*P*-value
**All Women Combined**						
All subjects	88,807	0.95 (0.87–1.02)	0.90 (0.82–0.98)	0.84 (0.71–0.97)	0.65 (0.56–0.75)	3.32 × 10^−10^
**By Menopausal Status**						
Premenopausal	19,262	0.96 (0.88–1.05)	0.91 (0.83–1.00)	0.78 (0.67–0.9.0)	0.44 (0.31–0.62)	9.91 × 10^−8^
Postmenopausal	42,424	0.96 (0.9–1.01)	0.91 (0.85–0.96)	0.88 (0.81–0.96)	0.57 (0.46–0.71)	1.88 × 10^−8^
Never HRT use	11,433	0.98 (0.87–1.09)	0.92 (0.80–1.03)	0.89 (0.75–1.04)	0.60 (0.38–0.90)	0.0097
Ever HRT use	10,967	0.93 (0.82–1.04)	0.86 (0.74–0.97)	0.84 (0.69–0.99)	0.47 (0.29–0.73)	0.0002
**By ER Status**						
ER-positive	69,556	0.98 (0.93–1.02)	0.93 (0.89–0.98)	0.90 (0.84–0.96)	0.68 (0.57–0.81)	2.74 × 10^−6^
ER-negative	49,770	1.01 (0.87–1.15)	0.95 (0.88–1.02)	0.91 (0.83–0.98)	0.45 (0.33–0.59)	3.41 × 10^−10^
**By PR Status**						
PR-positive	62,231	0.98 (0.93–1.02)	0.93 (0.87–0.98)	0.89 (0.82–0.95)	0.65 (0.53–0.78)	9.52 × 10^−7^
PR-negative	52,208	1.13 (1.01–1.25)	0.92 (0.86–0.98)	0.90(0.84–0.97)	0.47 (0.36–0.60)	2.84 × 10^−11^
**By ER/PR Status**						
ER/PR-positive	61,430	0.97 (0.92–1.02)	0.93 (0.87–0.98)	0.89 (0.82–0.95)	0.66 (0.55–0.8)	5.46 × 10^−6^
ER/PR-negative	28,855	0.93 (0.85–1.01)	0.90 (0.82–0.98)	0.80 (0.69–0.90)	0.42 (0.3–0.58)	7.19 × 10^−10^

ER, estrogen receptor; PR, progesterone receptor. Models were adjusted for age, first eight principal components, study sites, age at menarche, parity, use of contraceptive, use of hormone replacement therapy, breast feeding, and smoking status.

* BMI <25 is used as reference.

^†^ Results are presented for per 5 kg/m^2^ increase.

In pooled analysis of the BCAC data, 15 of the 84 SNPs analyzed in the study showed an inverse association with breast cancer risk, and one showed positive association with breast cancer risk at *p* < 0.05 (S9 and S10 Tables). In the DRIVE dataset, 12 of the 84 SNP were significantly inversely associated with breast cancer risk, including 9 SNPs that were also significant in the BCAC data (S8 and S10 Tables). When the datasets were combined, 17 SNPs showed an association with breast cancer risk at *p* < 0.05, and 16 of them showed an inverse association (Table 3 and S10 Table). Five of the associations remained statistically significant after adjusting for multiple comparisons (*p* < 0.0006 for 84 comparisons).

**Table 3:** Significant associations detected at *p* < 0.05 between breast cancer risk and BMI-related SNPs.

					BCAC*			GAME-ON DRIVE			Combined	
SNP	Chr	Position	Gene	Alleles	EAF	OR (95% CI)	*P*	EAF	OR (95% CI)	*P*	OR (95% CI)*	*P*
rs1558902	16	53803574	RABEP1(N)	A/T	0.41	0.93 (0.91–0.95)	2.77 × 10^−14^	0.68	0.95 (0.91–0.99)	0.008	0.93 (0.91–0.95)	3.63 × 10^−16^
rs713586	2	25158008	STXBP6(N)	C/T	0.47	0.94 (0.92–0.97)	1.82 × 10^−6^	0.48	0.96 (0.93–1.00)	0.03	0.95 (0.93–0.97)	3.19 × 10^−7^
rs7903146	10	114758349	NRXN3	C/T	0.72	0.96 (0.94–0.98)	7.01 × 10^−5^	0.70	0.96(0.92–1.00)	0.04	0.96 (0.94–0.98)	8.65 × 10^−6^
rs7599312	2	213413231	LMX1B(B,N)	G/A	0.72	0.96 (0.94–0.98)	0.0004	0.96	0.94(0.84–1.03)	0.17	0.96 (0.94–0.98)	0.0002
rs17024393	1	110154688	BDNF(B/M)	C/T	0.03	0.93 (0.87–0.98)	0.007	0.41	0.96 (0.92–0.99)	0.009	0.94 (0.91–0.97)	0.0003
rs2867125	2	622827	GNPDA2(N)	C/T	0.83	0.96 (0.94–0.99)	0.003	0.64	0.97 (0.94–1.00)	0.07	0.96 (0.94–0.99)	0.0008
rs2287019	19	46202172	LI NG02(D,N)	C/T	0.79	0.96 (0.93–0.99)	0.009	0.80	0.96 (0.92–1.00)	0.06	0.96 (0.94–0.99)	0.0010
rs3810291	19	47569003	CLIP1(N)	A/G	0.67	0.98 (0.95–1.00)	0.01	0.43	0.96 (0.92–0.99)	0.01	0.97 (0.95–0.99)	0.002
rs571312	18	57839769	NT5C2(N)	A/C	0.24	0.97 (0.95–1.00)	0.02	0.23	0.96 (0.92–1.00)	0.04	0.97 (0.95–0.99)	0.002
rs543874	1	177889480	ELAVL4(B,D,N,Q)	G/A	0.19	0.97 (0.95–1.00)	0.04	0.20	0.96 (0.92–1.00)	0.04	0.97 (0.95–0.99)	0.005
rs12401738	1	78446761	HIP1(B,N)	A/G	0.38	0.98 (0.96–1.00)	0.05	0.38	0.96 (0.93–1.00)	0.05	0.97 (0.96–0.99)	0.008
rs1528435	2	181550962	EHBP1(B,N)	T/C	0.62	0.97 (0.95–0.99)	0.01	0.63	0.98 (0.94–1.01)	0.22	0.97 (0.96–0.99)	0.008
rs2112347	5	75015242	PRKDI(N)	T/G	0.63	0.98 (0.96–1.00)	0.03	0.44	0.97 (0.94–1.00)	0.08	0.98 (0.96–0.99)	0.008
rs10733682	9	129460914	FUBPI(N)	A/G	0.49	0.97 (0.95–0.99)	0.009	0.47	0.99 (0.95–1.02)	0.41	0.98 (0.96–0.99)	0.01
rs13191362	6	163033350	GPRC5B(C/Q)	A/G	0.88	1.03 (1.00–1.06)	0.047	0.87	1.04 (0.98–1.09)	0.18	1.03 (1.01–1.06)	0.02
rs17405819	8	76806584	PRKD1(N)	T/C	0.69	0.97 (0.95–1.00)	0.02	0.69	0.99 (0.95–1.02)	0.5	0.98 (0.96–1.00)	0.02
rs3736485	15	51748610	CADM2	A/G	0.47	0.98 (0.96–1.00)	0.12	0.43	0.98 (0.94–1.01)	0.15	0.98 (0.96–1.00)	0.04

* Results are presented for per allele increase of BMI-related SNP. Chr, chromosome; EAF, effective allele frequency. BCAC models were adjusted for age, study, and first eight principal components.

Using data from BCAC, we conducted pooled analyses to evaluate the association of observed BMI with breast cancer risk by study design. Data from prospective cohort studies showed a positive association between observed BMI and breast cancer risk among postmenopausal women, while an inverse association was seen among premenopausal women (S11 Table). Data from nonprospective studies, however, showed an inverse association for both pre- and postmenopausal women. Additional adjustment for BMI-GS did not alter the association between observed BMI and breast cancer risk.

## Discussion

Utilizing data from two large consortia, we found in this large MR study a consistent inverse association between BMI predicted by GWAS-identified genetic variants and premenopausal breast cancer risk in all subgroups examined, which is qualitatively consistent with the majority of published epidemiologic studies using measured BMI, although our predicted association, a 46% reduction in risk per 5 kg/m^2^ increase in BMI, is larger than that estimated in observational studies using measured BMI [1,3,5,8,29]. Prominent hypotheses regarding the underlying cause of the association between higher BMI and decreased premenopausal breast cancer risk implicate more frequent anovulation, lower endogenous estrogen levels, and fewer breast cell divisions in obese women as compared to leaner women.

Our MR analyses demonstrate an inverse association between genetically predicted BMI and postmenopausal breast cancer risk, with a predicted effect similar to that seen in premenopausal women. In contrast, previous large observational studies indicate a 5%–15% increased risk for postmenopausal breast cancer per 5 kg/m^2^ increase in BMI [1,8]. In our analysis of prospective cohort studies included in BCAC, we observed a similar increase in breast cancer risk associated with observed BMI among postmenopausal women. However, this positive association was not found in the analysis of data from case-control studies included in BCAC, perhaps due to reverse causation. Because disease diagnosis and progress could change body weight, BMI measured after cancer diagnosis, which is done in most case-control studies, does not reflect usual or long-term BMI, and case-control studies are biased in evaluating the association of BMI and cancer risk. Because no BMI data from cases were used in our MR analyses, we have effectively overcome the possible influence of reverse causation in our study results from MR analyses.

The finding for an inverse association between BMI predicted using GWAS-identified SNPs and postmenopausal breast cancer risk differs from findings reported previously in studies using measured BMI, revealing a complex relationship of genetic determinants of BMI, weight gain, and breast cancer risk. A recent study found that a BMI-GS composed of 31 GWAS-identified SNPs (the majority of which are included in our study) was positively associated with annual weight gain between age 20 y and the time of the study baseline interview when participants were middle-aged [30]. On the other hand, this GS was related to a reduced weight in later adulthood. These results suggest that the genetic portion of BMI, as measured using the BMI-GS in our study, may reflect an early-life BMI.

Several studies found that early-life BMI was inversely associated with breast cancer risk, and this inverse association is consistent in premenopausal [31,32] and postmenopausal [31,33] women. It is possible that weight gain during later adulthood, not adult BMI per se, is related to increased postmenopausal breast cancer risk among overweight women as determined using measured BMI. However, we were unable to directly evaluate this hypothesis in our study because adult weight change was not consistently measured in the BCAC contributing studies. Furthermore, the SNPs used to construct the BMI-GS were identified from genetic association studies that included mostly middle-aged adults, and thus, they may not be able to measure weight gain in later adulthood adequately.

After menopause, the primary source of estrogen is formed in adipose tissue, [11,34] causing overweight and obese postmenopausal women to have higher circulating overall and free estradiol levels than their normal BMI counterparts. In premenopausal women, a high BMI is related to anovulatory menstrual cycles. Women with high BMI in both pre- and postmenopause may have lower lifetime estrogen exposure (and thus lower risk of breast cancer) than those who gain weight primarily after menopause. Additionally, measured BMI in postmenopausal women may be a surrogate breast cancer risk factor for adiposity-related changes occurring near or after menopause, such as age-associated slowing metabolism and inflammation associated with increased abdominal fat [35]. Previous investigations support the theory that adult weight gain is positively associated with postmenopausal breast cancer risk, and some investigators have suggested that weight gain may be a more important risk factor for postmenopausal woman than postmenopausal BMI [36,37]. Future research will be necessary to determine the potentially complicated causal mechanisms underlying the association between BMI and breast cancer risk for postmenopausal women.

In our study, we observed associations of high BMI-GS with early age at menarche, low prevalence of postmenopausal HT use, and high prevalence of cigarette smoking. It is known that high body weight is associated with an early age at menarche [38], and overweight women are more likely to smoke cigarettes regularly to reduce or maintain body weight [39,40]. Overweight women are less likely to use HT [41] (likely because their endogenous estrogen levels are higher than normal/underweight women, and thus, they are less likely to experience postmenopausal symptoms—the major reason for HT use). Therefore, it is most likely that the association of these variables with the BMI-GS is mediated through BMI, indicating that the association of the BMI-GS with these breast cancer risk factors does not violate the assumption of MR analyses in our study. Indeed, analyses without adjusting for these variables revealed a stronger association of BMI-GS with breast cancer risk than those with adjustments of these variables. Some of the BMI-associated variants may be associated with certain functions in the central nervous system [17], and these functions in turn are associated with BMI and perhaps other behaviors currently unknown to us. It is also possible that some of the BMI-associated SNPs may be related to other traits. However, we were unable to evaluate these hypotheses in our study. It would be interesting to further evaluate possible pleiotropic effects of BMI-GS in future large MR analyses with extensively measured environmental factors.

We evaluated whether postmenopausal HT use may modify the association between BMI-GS and breast cancer risk or whether the association may vary by tumor hormone receptor status. Unlike some conventional observational studies on observed BMI-postmenopausal breast cancer association [9,42], we did not find the association for BMI-GS to be modified by HT use. We found that the association between the BMI-GS and breast cancer risk was consistent across hormone receptor subtypes. Although ER-positive and ER-negative breast cancer are heterogeneous clinically, they do have a number of shared risk factors, such as age at menarche, benign breast disease, and family history [43].

Our study has certain limitations. To date, GWAS-identified SNPs represent a small, but statistically significant, portion of the explained variance of observed BMI—approximately 2.7% [17,44,45]. Nevertheless, the instrumental variable created in our study is sufficiently strong for conducting MR analyses [46]. Only summary statistics data were available from the DRIVE project, and thus, we were unable to perform analyses stratified by menopausal status and hormone receptor status. However, most of the subjects included in the DRIVE project were postmenopausal women, and the strength of the association between BMI-GS and breast cancer observed in BCAC and DRIVE consortia was similar.

Using data from approximately 146,000 women involved in two large consortia, we provide strong evidence of an inverse association between genetically predicted BMI and breast cancer risk for both premenopausal and postmenopausal women. The present study adds to the body of knowledge on the influence of body mass on breast cancer risk and points to further work required to elucidate the mechanisms responsible for the complex relationship between BMI and breast cancer risk. Our study, along with recent findings of an association of BMI-GS with weight gain in early adult life but weight loss in late adult life, suggests that weight gain later in adulthood may explain, at least partially, the positive association reported from previous studies between measured adult BMI and postmenopausal breast cancer risk, providing further support for lifestyle modification to reduce obesity as the primary prevention of breast cancer.

## Supporting Information

S1 Fig Egger regression funnel plot from the meta-analysis. The presence of funnel plot asymmetry indicates bias.
(EPS)

S1 Table Description of BCAC studies participating in this analysis. (DOCX)

S2 Table Characteristics of study participants included in the BCAC. (DOCX)

S3 Table Description of GAME-ON DRIVE Consortium studies participating in this analysis. (DOCX)

S4 Table GS computed for sensitivity analyses. (DOCX)

S5 Table Associations of the 84 SNPs with observed BMI in the BCAC. (DOCX)

S6 Table Associations of the weighted BMI-GS with traditional breast cancer risk factors adjusting for observed BMI. (DOCX)

S7 Table Association of genetically predicted BMI and breast cancer risk, stratified by age group. (DOCX)

S8 Table MR analysis of BMI and breast cancer risk in women using summary data from published BMI GWAS and DRIVE Breast Cancer GWAS. (DOCX)

S9 Table Associations of the 84 SNPs with breast cancer risk in the BCAC. (DOCX)

S10 Table Association of breast cancer risk with 84 BMI-related SNPs. (DOCX)

S11 Table The associations between observed BMI and breast cancer risk using BCAC data. (DOCX)

S1 Text Complete funding statement. (DOCX)

## Abbreviations

BCAC: Breast Cancer Association Consortium
BMI: body mass index
COGS: Collaborative Oncological Gene Environment Study
DRIVE: Discovery, Biology, and Risk of Inherited Variants in Breast Cancer
ER: estrogen receptor
GAME-ON: Genetic Associations and Mechanisms in Oncology
GIANT: Genetic Investigation of Anthropometric Traits
GS: genetic score
GWAS: genome-wide association studies
HRT: hormone replacement therapy
HT: hormone therapy
MR: Mendelian randomization
OR: odds ratio
SNP: single nucleotide polymorphism
