# Supplementary material for: Genetically Predicted Body Mass Index and Breast Cancer Risk: Mendelian Randomization Analyses of Data from 145,000 Women of European Descent
Source: PLoS Med. 2016 Aug 23;13(8):e1002105. doi: 10.1371/journal.pmed.1002105 (PMC4995025; doi:10.1371/journal.pmed.1002105)
Supplement: S2 Table — (DOCX) [file pmed.1002105.s003.docx]

| **S2 Table. Characteristics of study participants included in the Breast Cancer Association Consortium.** | | | | | | | | | | | | | | |
| --- | --- | --- | --- | --- | --- | --- | --- | --- | --- | --- | --- | --- | --- | --- |
| **Study*** | **Characteristics of cases** | | | | | | | |  | **Characteristics of controls** | | | | |
|  | **N** | **Age, year** | **Age at menarche** | **Height, cm** | **Menopause** | **Observed**  **BMI km/m^2^** | **ER+** | **PR+** |  | **N** | **Age, year** | **Age at menarche** | **Observed**  **BMI, km/m^2^** | **Menopause** |
|  |  | **(range)** | **year (range)** | **(range)** | **(%)** | **(range)** | **(%)** | **(%)** |  |  | **(range)** | **year (range)** | **(range)** | **(%)** |
| ABCFS | 790 | 40.4 (23-60) | 12.8 (9-17) | 164.1 (150-185) | 21 | 24.8 (15-43) | 63.6 | 71.1 |  | 551 | 41.9 (20-60) | 12.9 (9-18) | 25.0 (16-51) | 35.1 |
| ABCS | 1325 | 46.6 (20-83) | 13.1 (10-17) | 170.1 (150-190) | 61.9 | 24.6 (17-46) | 73.3 | 58.6 |  | 1429 | 47.7 (18-70) | 13.1 (9-19) | 25.1 (17-62) | 44.2 |
| BBCC | 564 | 60.8 (23-90) | 13.5 (8-18) | 164.6 (145-188) | 77.8 | 26.8 (13-53) | 84.7 | 71.8 |  | 458 | 57.1 (28-87) | 13.4 (10-21) | 25.4 (16-56) | 75.8 |
| BBCS | 1554 | 57.5 (28-84) | 12.6 (10-19) | 167.1 (163-170) | 100 | 22.5 (18-25) | 81.6 | 67.2 |  | 1397 | 45.0 (16-79) | 12.8 (8-17) | 26.5 (18-51) | 61 |
| BIGGS | 836 | 58.7 (32-93) | N/A | N/A | N/A | N/A | 76.3 | 75.7 |  | 719 | 65.6 (25-96) | N/A | N/A | N/A |
| BSUCH | 852 | 57.6 (25-88) | N/A | N/A | 62.1 | N/A | 76.4 | 69.4 |  | 954 | 57.3 (50-69) | N/A | N/A | N/A |
| CECILE | 1019 | 54.4 (25-74) | 12.9 (9-18) | 161.8 (134-180) | 60.6 | 24.5 (14-47) | 84.7 | 70.5 |  | 999 | 54.7 (25-74) | 13.1 (9-21) | 25.0 (14-55) | 63.7 |
| CGPS | 2901 | 61.8 (24-96) | 13.6 (8-22) | 165.7 (139-186) | 79.1 | 24.7 (14-58) | 84.3 | 64.7 |  | 4086 | 57.9 (21-90) | N/A | 25.0 (13-50) | 70.1 |
| CNIO-BCS | 902 | 57.4 (31-88) | 13.3 (9-19) | N/A | 76.8 | 16.4 (16-16) | 73.3 | 54.2 |  | 876 | 50.0 (23-86) | 12.9 (9-18) | 25.7 (10-44) | 75.9 |
| CTS | 68 | 55.8 (39-69) | 12.5 (10-17) | N/A | 66.7 | 25.5 (18-40) | 0 | 0 |  | 71 | 55.6 (33-74) | 12.5 (10-15) | 24.6 (18-50) | 70 |
| ESTHER | 478 | 60.6 (30-79) | 13.6 (9-19) | 163.3 (140-184) | 89 | 27.3 (17-48) | 75.6 | 66 |  | 502 | 62.3 (49-75) | 13.4 (9-18) | 27.6 (17-46) | 94.8 |
| GC-HBOC | 0 | N/A | N/A | N/A | N/A | N/A | N/A | N/A |  | 139 | 57.7 (46-68) | N/A | N/A | N/A |
| GENICA | 465 | 57.0 (25-80) | 13.4 (9-18) | 165.5 (148-184) | 72.1 | 25.6 (17-53) | 73.4 | 68 |  | 427 | 57.3 (24-80) | 13.7 (8-19) | 25.3 (18-52) | 72 |
| HEBCS | 1664 | 58.1 (25-96) | 13.3 (9-20) | 164.3 (148-183) | 66.6 | 25 (15-44) | 84.5 | 67.7 |  | 1234 | 40.9 (18-66) | N/A | N/A | N/A |
| HMBCS | 690 | 48.8 (16-82) | 13.8 (13-15) | N/A | 25 | N/A | 82.2 | N/A |  | 130 | 41.9 (22-71) | N/A | N/A | N/A |
| KARBAC | 722 | 60.9 (27-88) | 13.4 (9-18) | N/A | 66.1 | N/A | 84.3 | 76.3 |  | 662 | N/A | N/A | N/A | N/A |
| KBCP | 445 | 58.9 (23-92) | 13.8 (10-19) | 162.0 (145-178) | 70 | 26.5 (17-51) | 75.8 | 62.9 |  | 251 | 52.8 (26-77) | 13.7 (10-18 ) | 26.0 (17-43) | 55.9 |
| kConFab/AOCS | 613 | 53.9 (25-95) | 13.0 (8-95) | 164.1 (120-189) | 74.2 | 25.9 (15-49) | 73.3 | 69.9 |  | 897 | 57.8 (21-83) | 13.1 (8-19) | 26.5 (16-51) | 72.5 |
| LMBC | 2671 | 56.9 (21-94) | 13.3 (8-21) | 163.1 (135-198) | 65.2 | 25.7 (16-61) | 84.5 | 76.7 |  | 1388 | 43.9 (19-66) | N/A | 24.6 (16-56) | N/A |
| MARIE | 1818 | 62.5 (50-78) | 13.6 (9-20) | 164.4 (140-193) | 87.8 | 26.1 (16-50) | 77.2 | 65.4 |  | 1778 | 61.8 (49-75) | 13.6 (8-20) | 26.4 (16-53) | 90.1 |
| MBCSG | 488 | 49.4 (22-87) | 12.4 (9-16) | 163.5 (144-179) | 36.6 | 23.1 (17-33) | 78 | 70.2 |  | 400 | 42.3 (21-69) | N/A | N/A | N/A |
| MCBCS | 1862 | 57.1 (22-93) | 12.7 (9-19) | 165.3 (130-212) | 68.3 | 27.7 (16-57) | 83.4 | 74.1 |  | 1931 | 56.6 (21-91) | 12.8 (9-17) | 28.0 (16-63) | 70.4 |
| MCCS | 614 | 56.7 (37-70) | 13.1 (9-20) | 160.8 (142-181) | 73.3 | 26.7 (18-47) | 74.7 | 63.9 |  | 511 | 56.3 (38-70) | 12.9 (9-18) | 26.1 (16-48) | 75.2 |
| MEC | 731 | 59.8 (48-76) | N/A | N/A | N/A | N/A | 82.7 | 71.7 |  | 741 | 59.7 (45-76) | N/A | N/A | N/A |
| MTLGEBCS | 489 | 62.7 (50-77) | N/A | N/A | N/A | N/A | 86.8 | 73.8 |  | 436 | 61.6 (49-77) | N/A | N/A | N/A |
| NBCS | 22 | 58.8 (28-81) | N/A | 163.3 (163-164) | N/A | 22.7 (22-23) | 0 | 0 |  | 70 | 56.0 (32-71) | 13.6 (11-16) | 26.5 (19-43) | 78.9 |
| OBCS | 507 | 57.5 (28-92) | 13.4 (10-18) | N/A | 68.1 | N/A | 80.3 | 70.8 |  | 414 | 42.4 (18-66) | N/A | N/A | N/A |
| OFBCR | 1175 | 56.9 (26-84) | 12.6 (8-18) | 162.6 (99-185) | 78.5 | 25.9 (14-67) | 70.2 | 61.1 |  | 511 | 51.9 (25-69) | 12.6 (8-21) | 26.4 (16-52) | 56.2 |
| ORIGO | 357 | 57.2 (24-84) | 13.2 (9-19) | N/A | 75.9 | 25.6 (18-45) | 75.1 | 62.1 |  | 327 | N/A | N/A | N/A | N/A |
| PBCS | 519 | 56.6 (27-76) | 13.6 (9-21) | 162.1 (145-176) | 75.3 | 27.2 (16-47) | 100 | 69.1 |  | 424 | 56.3 (33-75) | 13.7 (8-19) | 28.0 (16-45) | 71.2 |
| pKARMA | 5434 | 63.1 (26-87) | 13.2 (8-22) | 165.9 (120-189) | 75.2 | N/A | 84 | 69.6 |  | 5537 | 53.9 (22-87) | 13.1 (8-25) | N/A | 53.3 |
| RBCS | 664 | N/A | 13.1 (10-18) | N/A | N/A | N/A | 73.8 | 64.4 |  | 699 | N/A | N/A | N/A | N/A |
| SASBAC | 1163 | 63.4 (50-75) | 13.5 (9-18) | 164.4 (147-182) | 99.9 | 25.6 (16-50) | 82.2 | 71.1 |  | 1378 | 63.3 (49-76) | 13.5 (8-21) | 25.6 (16-66) | 99.6 |
| SBCS | 843 | 63.0 (29-96) | 13.1 (9-20) | 162.2 (145-183) | 66.4 | 26.6 (16-47) | 78.2 | 58.4 |  | 848 | 57.5 (45-78) | 13.0 (9-18) | 27.0 (18-68) | 68.3 |
| SEARCH | 9347 | 56.1 (25-79) | 12.8 (8-19) | 163.3 (124-196) | 54.1 | 26.4 (14-59) | 81.4 | 70.3 |  | 8069 | 57.8 (28-80) | 12.9 (8-21) | 26.4 (17-53) | 70.3 |
| SKKDKFZS | 136 | 58.8 (22-87) | 13.8 (11-19) | 164.3 (145-185) | 72.6 | 26.2 (18-41) | 0 | 0 |  | 29 | 60.7 (46-68) | N/A | N/A | N/A |
| SZBCS | 365 | 58.1 (27-90) | N/A | N/A | 57.1 | N/A | 73.3 | 75.2 |  | 315 | 57.4 (29-82) | N/A | N/A | N/A |
| TNBCC | 756 | N/A | N/A | N/A | N/A | N/A | 0 | 0 |  | 424 | 55.4 (23-92) | N/A | N/A | N/A |
| UKBGS | 476 | 54.4 (23-82) | 12.7 (8-17) | 164.2 (145-183) | 64.2 | 25.6 (17-42) | 81.4 | 68.2 |  | 470 | 54.2 (23-82) | 12.6 (8-17) | 25.8 (18-49) | 64.2 |
| N/A=no data were available. | | |  |  |  |  |  |  |  |  |  |  |  |  |
| *The GC-HBOC from Germany only included controls and this study was combined with the SKKDKFZS from Germany in our analysis. | | | | | | | | | | | |  |  |  |
