# Supplementary material for: Genetically Predicted Body Mass Index and Breast Cancer Risk: Mendelian Randomization Analyses of Data from 145,000 Women of European Descent
Source: PLoS Med. 2016 Aug 23;13(8):e1002105. doi: 10.1371/journal.pmed.1002105 (PMC4995025; doi:10.1371/journal.pmed.1002105)
Supplement: S3 Table — (DOCX) [file pmed.1002105.s004.docx]

| **S3 Table. Description of GAME-ON DRIVE Consortium studies participating in this analysis.** | | | | | | |
| --- | --- | --- | --- | --- | --- | --- |
| **Study (Reference)** | **Country** | **Case Ascertainment** | **Control Ascertainment** | **Genotyping platform** | **Cases** | **Controls** |
| ABCFS/kConFab [[1](#_ENREF_1)] | Australia | Recruitment through cancer registries in Victoria and New South Wales | Recruitment from the electoral rolls in Melbourne and Sydney matched to cases by age in-5 year categories | Illumina 610k | 282 | 285 |
| BBCS [[4](#_ENREF_4)] | UK | Recruitment through cancer registries and clinics in the UK, predominantly bilateral cases | WTCCC2: 1958 Birth Cohort + UK National Blood Service | Illumina 370k (cases) Illumina 1.2M (controls) | 1609 | 5190 |
| GC-HBOC [[12](#_ENREF_12)] | Germany | BRCA1/2 mutation negative cases from University Clinics in Cologne and Munich | KORA (Cooperative Health Research in the Region Augsburg) | Affymetrix 5.0k (cases) Affymetrix 6.0k (controls) | 634 | 477 |
| MARIE [[20](#_ENREF_20)] | Germany | Random sample of cases from the MARIE study, but restricted to ductal and lobular carcinomas and oversampled for lobular (about 2:1) | KORA (Cooperative Health Research in the Region Augsburg) | Illumina 370k (cases) Illumina 550k (controls) | 708 | 470 |
| HEBCS [[14](#_ENREF_14)] [[39](#_ENREF_39)] | Finland | Unselected cases plus additional familial cases from Helsinki University Central Hospital | Population Controls from the NordicDB, a Nordic pool and portal for genome-wide control data | llumina 550k + 610k (cases) Illumina 370k (controls) | 810 | 1012 |
| SASBAC [[14](#_ENREF_14)] | Sweden | Population- based case control study of postmenopausal women | Population- based controls frequency matched by age to cases | Illumina 317k+240k (cases) Illumina 550k (controls) | 790 | 756 |
| UK2 [[40](#_ENREF_40)] | UK | UK cancer genetics clinics + oncology clinics | WTCCC2: 1958 Birth Cohort + UK National Blood Service | Illumina 670k (cases) Illumina 1.2M (controls) | 3628 | 5190 |
| DFBBCS [[41](#_ENREF_41)] | Netherlands | BRCA1/2 mutation negative familial bilateral breast cancer patients selected from five clinical genetics centers; Erasmus University Medical Center/Daniel den Hoed, The Netherlands Cancer Institute, Leiden University Medical Center, University Medical Center Utrecht, and VU University Medical Center. | Controls were from the Rotterdam study, and are 55 years or older at the time of inclusion. For this study females were selected and breast cancer cases were excluded. | Illumina 610k (cases) Illumina 550k (controls) | 464 | 3265 |
| BPC3 [[42](#_ENREF_42)] | US/Europe | Estrogen Receptor negative cases from population based cohorts within the Breast and Prostate cancer cohort consortium (BPC3) | Individually matched within cohorts in BPC3 | Illumina 660k+550K+317k | 2188 | 25519 |
| Early-onset Breast Cancer GWAS [[43](#_ENREF_43)] | US/Europe/ Australia | Population-based subjects were recruited from eight sites, some of which oversampled cases with a personal or family history. Eligible cases were non-Hispanic White women diagnosed with invasive breast cancer when 51 years or younger and not known to carry pathogenic mutations in BRCA1 or BRCA2. | Eligible controls were non-hispanic white women aged 20-51 years without a history of breast cancer, who were identified largely by random-digit dialing. | Illumina 610k + Cyto 12) | 3523 | 2702 |
| SardiNIA (N/A) | Italy | N/A | N/A | Affymetrix 500k (cases) Affymetrix 6.0k (controls) | 1367 | 1659 |
| GAME-ON=Genetic Associations and Mechanisms in Oncology. DRIVE=Discovery, Biology, and Risk of Inherited Variants in Breast Cancer. N/A=no data were available. | | | | | | |

**References for S1 Table and S3 Table**

1. Dite, G.S., et al., *Familial risks, early-onset breast cancer, and BRCA1 and BRCA2 germline mutations.* J Natl Cancer Inst, 2003. **95**(6): p. 448-57.

2. Schmidt, M.K., et al., *Breast cancer survival and tumor characteristics in premenopausal women carrying the CHEK2*1100delC germline mutation.* J Clin Oncol, 2007. **25**(1): p. 64-9.

3. Schrauder, M., et al., *Single nucleotide polymorphism D1853N of the ATM gene may alter the risk for breast cancer.* J Cancer Res Clin Oncol, 2008. **134**(8): p. 873-82.

4. Fletcher, O., et al., *Inconsistent association between the STK15 F31I genetic polymorphism and breast cancer risk.* J Natl Cancer Inst, 2006. **98**(14): p. 1014-8.

5. Colleran, G., et al., *The TGFBR1*6A/9A polymorphism is not associated with differential risk of breast cancer.* Breast Cancer Res Treat, 2010. **119**(2): p. 437-42.

6. Yang, R., et al., *Genetic variants within miR-126 and miR-335 are not associated with breast cancer risk.* Breast Cancer Res Treat, 2011. **127**(2): p. 549-54.

7. Villeneuve, S., et al., *Breast cancer risk by occupation and industry: analysis of the CECILE study, a population-based case-control study in France.* Am J Ind Med, 2011. **54**(7): p. 499-509.

8. Weischer, M., et al., *Increased risk of breast cancer associated with CHEK2*1100delC.* J Clin Oncol, 2007. **25**(1): p. 57-63.

9. Milne, R.L., et al., *ERCC4 associated with breast cancer risk: a two-stage case-control study using high-throughput genotyping.* Cancer Res, 2006. **66**(19): p. 9420-7.

10. Bernstein, L., et al., *High breast cancer incidence rates among California teachers: results from the California Teachers Study (United States).* Cancer Causes Control, 2002. **13**(7): p. 625-35.

11. Widschwendter, M., et al., *Epigenotyping in peripheral blood cell DNA and breast cancer risk: a proof of principle study.* PLoS One, 2008. **3**(7): p. e2656.

12. Frank, B., et al., *Association of the CASP10 V410I variant with reduced familial breast cancer risk and interaction with the CASP8 D302H variant.* Carcinogenesis, 2006. **27**(3): p. 606-9.

13. Justenhoven, C., et al., *The CYP1B1_1358_GG genotype is associated with estrogen receptor-negative breast cancer.* Breast Cancer Res Treat, 2008. **111**(1): p. 171-7.

14. Li, J., et al., *A combined analysis of genome-wide association studies in breast cancer.* Breast Cancer Res Treat, 2011. **126**(3): p. 717-27.

15. Bogdanova, N.V., et al., *High frequency and allele-specific differences of BRCA1 founder mutations in breast cancer and ovarian cancer patients from Belarus.* Clin Genet, 2010. **78**(4): p. 364-72.

16. Margolin, S., et al., *BRCA1 mutations in a population-based study of breast cancer in Stockholm County.* Genet Test, 2004. **8**(2): p. 127-32.

17. Hartikainen, J.M., et al., *An autosome-wide scan for linkage disequilibrium-based association in sporadic breast cancer cases in eastern Finland: three candidate regions found.* Cancer Epidemiol Biomarkers Prev, 2005. **14**(1): p. 75-80.

18. Beesley, J., et al., *Association between single-nucleotide polymorphisms in hormone metabolism and DNA repair genes and epithelial ovarian cancer: results from two Australian studies and an additional validation set.* Cancer Epidemiol Biomarkers Prev, 2007. **16**(12): p. 2557-65.

19. De Maeyer, L., et al., *Does estrogen receptor-negative/progesterone receptor-positive breast carcinoma exist?* Journal of Clinical Oncology, 2008. **26**(2): p. 335-336.

20. Flesch-Janys, D., et al., *Risk of different histological types of postmenopausal breast cancer by type and regimen of menopausal hormone therapy.* Int J Cancer, 2008. **123**(4): p. 933-41.

21. Catucci, I., et al., *SNPs in ultraconserved elements and familial breast cancer risk.* Carcinogenesis, 2009. **30**(3): p. 544-5; author reply 546.

22. Olson, J.E., et al., *A comprehensive examination of CYP19 variation and risk of breast cancer using two haplotype-tagging approaches.* Breast Cancer Research and Treatment, 2007. **102**(2): p. 237-247.

23. Giles, G.G. and D.R. English, *The Melbourne Collaborative Cohort Study.* IARC Sci Publ, 2002. **156**: p. 69-70.

24. Kolonel, L.N., et al., *A multiethnic cohort in Hawaii and Los Angeles: baseline characteristics.* Am J Epidemiol, 2000. **151**(4): p. 346-57.

25. Michailidou, K., et al., *Large-scale genotyping identifies 41 new loci associated with breast cancer risk.* Nat Genet, 2013. **45**(4): p. 353-61, 361e1-2.

26. Nordgard, S.H., et al., *Genome-wide analysis identifies 16q deletion associated with survival, molecular subtypes, mRNA expression, and germline haplotypes in breast cancer patients.* Genes Chromosomes Cancer, 2008. **47**(8): p. 680-96.

27. Erkko, H., et al., *A recurrent mutation in PALB2 in Finnish cancer families.* Nature, 2007. **446**(7133): p. 316-9.

28. John, E.M., et al., *The Breast Cancer Family Registry: an infrastructure for cooperative multinational, interdisciplinary and translational studies of the genetic epidemiology of breast cancer.* Breast Cancer Res, 2004. **6**(4): p. R375-89.

29. Huijts, P.E., et al., *Clinical correlates of low-risk variants in FGFR2, TNRC9, MAP3K1, LSP1 and 8q24 in a Dutch cohort of incident breast cancer cases.* Breast Cancer Res, 2007. **9**(6): p. R78.

30. Garcia-Closas, M., et al., *Established breast cancer risk factors by clinically important tumour characteristics.* Br J Cancer, 2006. **95**(1): p. 123-9.

31. Easton, D.F., et al., *Genome-wide association study identifies novel breast cancer susceptibility loci.* Nature, 2007. **447**(7148): p. 1087-U7.

32. Wedren, S., et al., *Oestrogen receptor alpha gene haplotype and postmenopausal breast cancer risk: a case control study.* Breast Cancer Research, 2004. **6**(4): p. R437-R449.

33. MacPherson, G., et al., *Association of a common variant of the CASP8 gene with reduced risk of breast cancer.* J Natl Cancer Inst, 2004. **96**(24): p. 1866-9.

34. Lesueur, F., et al., *Allelic association of the human homologue of the mouse modifier Ptprj with breast cancer.* Hum Mol Genet, 2005. **14**(16): p. 2349-56.

35. Rashid, M.U., et al., *German populations with infrequent CHEK2*1100delC and minor associations with early-onset and familial breast cancer.* Eur J Cancer, 2005. **41**(18): p. 2896-903.

36. Jakubowska, A., et al., *Do BRCA1 modifiers also affect the risk of breast cancer in non-carriers?* European Journal of Cancer, 2009. **45**(5): p. 837-842.

37. Stevens, K.N., et al., *Common breast cancer susceptibility loci are associated with triple-negative breast cancer.* Cancer Res, 2011. **71**(19): p. 6240-9.

38. Swerdlow, A.J., et al., *The Breakthrough Generations Study: design of a long-term UK cohort study to investigate breast cancer aetiology.* Br J Cancer, 2011. **105**(7): p. 911-7.

39. Leu, M., et al., *NordicDB: a Nordic pool and portal for genome-wide control data.* Eur J Hum Genet, 2010. **18**(12): p. 1322-6.

40. Turnbull, C., et al., *Genome-wide association study identifies five new breast cancer susceptibility loci.* Nat Genet, 2010. **42**(6): p. 504-7.

41. Hofman, A., et al., *The Rotterdam Study: 2010 objectives and design update.* Eur J Epidemiol, 2009. **24**(9): p. 553-72.

42. Siddiq, A., et al., *A meta-analysis of genome-wide association studies of breast cancer identifies two novel susceptibility loci at 6q14 and 20q11.* Human Molecular Genetics, 2012. **21**(24): p. 5373-5384.

43. Ahsan, H., et al., *A Genome-wide Association Study of Early-Onset Breast Cancer Identifies PFKM as a Novel Breast Cancer Gene and Supports a Common Genetic Spectrum for Breast Cancer at Any Age.* Cancer Epidemiology Biomarkers & Prevention, 2014. **23**(4): p. 658-669.
