# Supplementary material for: Genetically Predicted Body Mass Index and Breast Cancer Risk: Mendelian Randomization Analyses of Data from 145,000 Women of European Descent
Source: PLoS Med. 2016 Aug 23;13(8):e1002105. doi: 10.1371/journal.pmed.1002105 (PMC4995025; doi:10.1371/journal.pmed.1002105)
Supplement: S4 Table — (DOCX) [file pmed.1002105.s005.docx]

| **S4 Table. Genetic scores (GS) computed for sensitivity analyses.** | | | | |
| --- | --- | --- | --- | --- |
| **Genetic score** | **SNPs** | **Weighting method** | **Formula** | **Mean (range)** |
| GS (weighted) | 84 | Weight using regression coefficient from published GWAS | GS= ($\sum_{1}^{84} \beta_{i}{SNP}_{i}$) | 15.1(10.7-20) |
| GS (no weight) | 84 | No weight | GS=($\sum_{1}^{84} {SNP}_{i}$) | 78(53-100) |
| GS = genetic score. SNP = single-nucleotide polymorphism. | | | | |
