# Supplementary material for: Genetically Predicted Body Mass Index and Breast Cancer Risk: Mendelian Randomization Analyses of Data from 145,000 Women of European Descent
Source: PLoS Med. 2016 Aug 23;13(8):e1002105. doi: 10.1371/journal.pmed.1002105 (PMC4995025; doi:10.1371/journal.pmed.1002105)
Supplement: S5 Table — (DOCX) [file pmed.1002105.s006.docx]

| **S5 Table. Associations of the 84 SNPs with observed BMI in the Breast Cancer Association Consortium (pooled analysis)..** | | | | | | | | | | | | |
| --- | --- | --- | --- | --- | --- | --- | --- | --- | --- | --- | --- | --- |
|  |  |  |  | **Published GWAS** | | | |  | **Controls in BCAC** | | | |
|  |  |  |  | **(N=249,796)** | | | |  | **(N=22,084)** | | | |
| id | Chr | Position | Alleles | EAF | beta | SE | P |  | EAF | beta | SE | P |
| rs1558902 | 16 | 53803574 | A/T | 0.42 | 0.39 | 0.02 | 4.8×10^-120^ |  | 0.41 | 0.33 | 0.04 | 3.78×10^-13^ |
| rs571312 | 18 | 57839769 | A/C | 0.24 | 0.23 | 0.03 | 6.43×10^-42^ |  | 0.24 | 0.27 | 0.05 | 1.62×10^-07^ |
| rs543874 | 1 | 177889480 | G/A | 0.19 | 0.22 | 0.03 | 3.56×10-^23^ |  | 0.19 | 0.25 | 0.06 | 5.03×10^-06^ |
| rs1808579 | 18 | 21104888 | C/T | 0.53 | 0.17 | 0.03 | 4.17×10^-08^ |  | 0.53 | 0.19 | 0.05 | 3.63×10^-05^ |
| rs205262 | 6 | 345631168 | G/A | 0.27 | 0.22 | 0.04 | 1.75×10^-10^ |  | 0.27 | 0.18 | 0.05 | 0.0002 |
| rs10968576 | 9 | 28414339 | G/A | 0.31 | 0.11 | 0.02 | 2.65×10^-13^ |  | 0.31 | 0.17 | 0.05 | 0.0003 |
| rs7359397 | 16 | 28885659 | T/C | 0.40 | 0.15 | 0.02 | 1.88×10^-20^ |  | 0.39 | 0.16 | 0.05 | 0.0006 |
| rs3817334 | 11 | 47650993 | T/C | 0.41 | 0.06 | 0.02 | 1.59×10^-12^ |  | 0.41 | 0.18 | 0.05 | 0.0007 |
| rs2867125 | 2 | 622827 | C/T | 0.83 | 0.31 | 0.03 | 2.77×10^-49^ |  | 0.83 | 0.20 | 0.06 | 0.0008 |
| rs1555543 | 1 | 96944797 | C/A | 0.59 | 0.06 | 0.02 | 3.68×10^-10^ |  | 0.60 | 0.17 | 0.05 | 0.001 |
| rs99259168 | 16 | 31129895 | A/G | 0.62 | 0.19 | 0.03 | 8.11×10^-10^ |  | 0.63 | 0.15 | 0.05 | 0.001 |
| rs10938397 | 4 | 45182527 | G/A | 0.43 | 0.18 | 0.02 | 3.78×10^-31^ |  | 0.43 | 0.14 | 0.04 | 0.001 |
| rs987237 | 6 | 50803050 | G/A | 0.18 | 0.13 | 0.03 | 2.90×10^-20^ |  | 0.18 | 0.18 | 0.06 | 0.002 |
| rs4787491 | 16 | 30015337 | G/A | 0.51 | 0.22 | 0.04 | 2.70×10^-8^ |  | 0.54 | 0.14 | 0.05 | 0.002 |
| rs10132280 | 14 | 25928179 | C/A | 0.68 | 0.23 | 0.03 | 1.14×10^-11^ |  | 0.69 | 0.16 | 0.05 | 0.002 |
| rs7243357 | 18 | 56883319 | T/G | 0.81 | 0.22 | 0.04 | 3.86×10^-8^ |  | 0.83 | 0.19 | 0.06 | 0.002 |
| rs713586 | 2 | 25158008 | C/T | 0.47 | 0.14 | 0.02 | 6.17×10^-22^ |  | 0.47 | 0.17 | 0.06 | 0.003 |
| rs10150332 | 14 | 799369168 | C/T | 0.21 | 0.13 | 0.03 | 2.75×10^-11^ |  | 0.21 | 0.16 | 0.05 | 0.003 |
| rs7138803 | 12 | 50247468 | A/G | 0.38 | 0.12 | 0.02 | 1.82×10^-17^ |  | 0.39 | 0.13 | 0.05 | 0.005 |
| rs1514175 | 1 | 749911684 | A/G | 0.43 | 0.07 | 0.02 | 8.16×10^-14^ |  | 0.42 | 0.13 | 0.05 | 0.006 |
| rs11057405 | 12 | 122781897 | G/A | 0.90 | 0.31 | 0.06 | 2.02×10^-8^ |  | 0.90 | 0.24 | 0.09 | 0.007 |
| rs16851483 | 3 | 141275436 | T/G | 0.07 | 0.48 | 0.08 | 3.55×10^-10^ |  | 0.06 | 0.25 | 0.09 | 0.007 |
| rs12885454 | 14 | 29736838 | C/A | 0.168 | 0.21 | 0.03 | 1.94×10^-10^ |  | 0.168 | 0.12 | 0.05 | 0.008 |
| rs9400239 | 6 | 108977663 | C/T | 0.69 | 0.19 | 0.03 | 1.61×10^-8^ |  | 0.70 | 0.13 | 0.05 | 0.010 |
| rs1167827 | 7 | 75163169 | G/A | 0.55 | 0.20 | 0.03 | 6.33×10^-10^ |  | 0.57 | 0.12 | 0.04 | 0.01 |
| rs4836133 | 5 | 124332103 | A/C | 0.48 | 0.07 | 0.02 | 1.97×10^-9^ |  | 0.52 | 0.11 | 0.04 | 0.01 |
| rs3810291 | 19 | 47569003 | A/G | 0.67 | 0.09 | 0.02 | 1.168×10^-12^ |  | 0.67 | 0.12 | 0.05 | 0.01 |
| rs887912 | 2 | 59302877 | T/C | 0.29 | 0.10 | 0.02 | 1.79×10^-12^ |  | 0.29 | 0.12 | 0.05 | 0.01 |
| rs2112347 | 5 | 75015242 | T/G | 0.63 | 0.10 | 0.02 | 2.17×10^-13^ |  | 0.63 | 0.11 | 0.05 | 0.02 |
| rs2241423 | 15 | 68086838 | G/A | 0.78 | 0.13 | 0.02 | 1.19×10^-18^ |  | 0.77 | 0.12 | 0.05 | 0.02 |
| rs17405819 | 8 | 76806584 | T/C | 0.70 | 0.22 | 0.03 | 2.07×10^-11^ |  | 0.69 | 0.11 | 0.05 | 0.02 |
| rs758747 | 16 | 3627358 | T/C | 0.27 | 0.23 | 0.04 | 7.47×10^-10^ |  | 0.27 | 0.11 | 0.05 | 0.02 |
| rs2287019 | 19 | 46202172 | C/T | 0.80 | 0.15 | 0.03 | 1.88×10^-16^ |  | 0.79 | 0.15 | 0.07 | 0.03 |
| rs206936 | 6 | 34302869 | G/A | 0.21 | 0.06 | 0.02 | 3.02×10^-8^ |  | 0.20 | 0.12 | 0.06 | 0.03 |
| rs2836754 | 21 | 40291740 | C/T | 0.60 | 0.17 | 0.03 | 1.61×10^-8^ |  | 0.168 | 0.10 | 0.05 | 0.03 |
| rs17024393 | 1 | 110154688 | C/T | 0.04 | 0.66 | 0.09 | 7.03×10^-14^ |  | 0.03 | 0.27 | 0.12 | 0.03 |
| rs2815752 | 1 | 72812440 | A/G | 0.61 | 0.13 | 0.02 | 1.61×10^-22^ |  | 0.61 | 0.10 | 0.05 | 0.04 |
| rs2890652 | 2 | 142959931 | C/T | 0.18 | 0.09 | 0.03 | 1.35×10^-10^ |  | 0.17 | 0.13 | 0.06 | 0.04 |
| rs1000940 | 17 | 5283252 | G/A | 0.32 | 0.19 | 0.03 | 1.28×10^-8^ |  | 0.30 | 0.10 | 0.05 | 0.04 |
| rs2080454 | 16 | 49062590 | C/A | 0.41 | 0.17 | 0.03 | 8.60×10^-9^ |  | 0.43 | 0.10 | 0.05 | 0.06 |
| rs2365389 | 3 | 612316862 | C/T | 0.58 | 0.20 | 0.03 | 1.63×10^-10^ |  | 0.58 | 0.08 | 0.05 | 0.08 |
| rs11191560 | 10 | 104869038 | C/T | 0.09 | 0.31 | 0.05 | 8.45×10^-9^ |  | 0.09 | 0.14 | 0.08 | 0.08 |
| rs3849570 | 3 | 81792112 | A/C | 0.36 | 0.19 | 0.03 | 2.60×10^-8^ |  | 0.33 | 0.09 | 0.05 | 0.10 |
| rs13107325 | 4 | 103188709 | T/C | 0.07 | 0.19 | 0.04 | 1.50×10^-13^ |  | 0.07 | 0.16 | 0.10 | 0.10 |
| rs4771122 | 13 | 28020180 | G/A | 0.24 | 0.09 | 0.03 | 9.48×10^-10^ |  | 0.25 | 0.09 | 0.05 | 0.10 |
| rs4929949 | 11 | 8604593 | C/T | 0.52 | 0.06 | 0.02 | 2.80×10^-9^ |  | 0.51 | 0.07 | 0.05 | 0.13 |
| rs9374842 | 6 | 120185665 | T/C | 0.74 | 0.23 | 0.04 | 2.67×10^-8^ |  | 0.77 | 0.08 | 0.06 | 0.14 |
| rs12401738 | 1 | 78446761 | A/G | 0.35 | 0.21 | 0.03 | 1.15×10^-10^ |  | 0.38 | 0.07 | 0.05 | 0.19 |
| rs13191362 | 6 | 163033350 | A/G | 0.88 | 0.28 | 0.05 | 7.34×10^-9^ |  | 0.88 | 0.09 | 0.07 | 0.19 |
| rs14412168 | 13 | 79580919 | A/G | 0.61 | 0.17 | 0.03 | 2.96×10^-8^ |  | 0.17 | 0.07 | 0.06 | 0.22 |
| rs17724992 | 19 | 18454825 | A/G | 0.75 | 0.19 | 0.04 | 3.42×10^-8^ |  | 0.75 | 0.06 | 0.05 | 0.22 |
| rs12444979 | 16 | 19933600 | C/T | 0.87 | 0.17 | 0.03 | 2.91×10^-21^ |  | 0.86 | 0.08 | 0.06 | 0.23 |
| rs977747 | 1 | 47684677 | T/G | 0.40 | 0.17 | 0.03 | 2.18×10^-8^ |  | 0.39 | 0.05 | 0.05 | 0.24 |
| rs7715256 | 5 | 153537893 | G/T | 0.42 | 0.17 | 0.03 | 8.85×10^-9^ |  | 0.42 | 0.05 | 0.04 | 0.24 |
| rs91681123 | 7 | 93197732 | C/G | 0.43 | 0.29 | 0.05 | 2.08×10^-10^ |  | 0.41 | 0.05 | 0.04 | 0.25 |
| rs16877694 | 9 | 111932342 | C/T | 0.37 | 0.17 | 0.03 | 2.67×10^-8^ |  | 0.36 | 0.05 | 0.05 | 0.26 |
| rs11688816 | 2 | 63053048 | G/A | 0.53 | 0.17 | 0.03 | 1.89×10^-8^ |  | 0.53 | 0.05 | 0.04 | 0.26 |
| rs4740619 | 9 | 15634326 | T/C | 0.54 | 0.18 | 0.03 | 4.56×10^-9^ |  | 0.54 | 0.05 | 0.05 | 0.28 |
| rs11847697 | 14 | 30515112 | T/C | 0.04 | 0.17 | 0.05 | 5.76×10^-11^ |  | 0.06 | 0.15 | 0.14 | 0.28 |
| rs71168727 | 15 | 73093991 | T/C | 0.67 | 0.19 | 0.03 | 3.92×10^-9^ |  | 0.68 | 0.05 | 0.05 | 0.31 |
| rs13078807 | 3 | 85884150 | G/A | 0.20 | 0.10 | 0.02 | 3.94×10^-11^ |  | 0.19 | 0.06 | 0.06 | 0.33 |
| rs7239883 | 18 | 40147671 | G/A | 0.39 | 0.23 | 0.04 | 1.51×10^-8^ |  | 0.40 | 0.05 | 0.05 | 0.33 |
| rs107676168 | 11 | 27725986 | A/T | 0.78 | 0.19 | 0.03 | 4.69×10^-26^ |  | 0.78 | 0.05 | 0.06 | 0.38 |
| rs7599312 | 2 | 213413231 | G/A | 0.72 | 0.22 | 0.03 | 1.17×10^-10^ |  | 0.72 | 0.04 | 0.05 | 0.40 |
| rs16907751 | 8 | 81375457 | C/T | 0.91 | 0.47 | 0.09 | 3.89×10^-8^ |  | 0.89 | 0.07 | 0.08 | 0.42 |
| rs1528435 | 2 | 181550962 | T/C | 0.63 | 0.18 | 0.03 | 1.20×10^-8^ |  | 0.62 | 0.04 | 0.05 | 0.44 |
| rs29941 | 19 | 34309532 | G/A | 0.67 | 0.06 | 0.02 | 3.01×10-9 |  | 0.68 | 0.04 | 0.05 | 0.45 |
| rs9914578 | 17 | 2005136 | G/C | 0.23 | 0.20 | 0.04 | 2.07×10-8 |  | 0.20 | 0.04 | 0.06 | 0.47 |
| rs11583200 | 1 | 50559820 | C/T | 0.40 | 0.18 | 0.03 | 1.48×10-8 |  | 0.39 | -0.03 | 0.05 | 0.50 |
| rs37316885 | 15 | 51748610 | A/G | 0.45 | 0.18 | 0.03 | 7.41×10-9 |  | 0.47 | 0.03 | 0.05 | 0.51 |
| rs2075650 | 19 | 45395619 | A/G | 0.85 | 0.26 | 0.05 | 1.25×10^-8^ |  | 0.85 | 0.04 | 0.07 | 0.60 |
| rs2176598 | 11 | 438168278 | T/C | 0.25 | 0.20 | 0.04 | 2.97×10^-8^ |  | 0.25 | 0.03 | 0.05 | 0.61 |
| rs9816226 | 3 | 185834499 | T/A | 0.82 | 0.14 | 0.03 | 1.69×10^-18^ |  | 0.82 | 0.03 | 0.06 | 0.61 |
| rs17203016 | 2 | 208255518 | G/A | 0.20 | 0.21 | 0.04 | 3.41×10^-8^ |  | 0.20 | -0.03 | 0.06 | 0.63 |
| rs2820292 | 1 | 201784287 | C/A | 0.56 | 0.20 | 0.03 | 1.83×10^-10^ |  | 0.56 | 0.02 | 0.05 | 0.70 |
| rs492400 | 2 | 219349752 | C/T | 0.42 | 0.24 | 0.04 | 6.78×10^-9^ |  | 0.41 | 0.02 | 0.05 | 0.74 |
| rs7899106 | 10 | 87410904 | G/A | 0.05 | 0.40 | 0.07 | 2.96×10^-8^ |  | 0.05 | 0.03 | 0.11 | 0.75 |
| rs1928295 | 9 | 120378483 | T/C | 0.55 | 0.19 | 0.03 | 7.91×10^-10^ |  | 0.55 | 0.01 | 0.05 | 0.78 |
| rs657452 | 1 | 49589847 | A/G | 0.39 | 0.23 | 0.03 | 5.48×10^-13^ |  | 0.37 | -0.01 | 0.05 | 0.79 |
| rs10733682 | 9 | 129460914 | A/G | 0.48 | 0.17 | 0.03 | 1.83×10^-8^ |  | 0.49 | 0.01 | 0.05 | 0.83 |
| rs17001654 | 4 | 77129568 | G/C | 0.15 | 0.31 | 0.05 | 7.76×10^-9^ |  | 0.16 | 0.01 | 0.07 | 0.86 |
| rs17094222 | 10 | 102395440 | C/T | 0.21 | 0.25 | 0.04 | 5.94×10^-11^ |  | 0.22 | -0.01 | 0.06 | 0.88 |
| rs2176040 | 2 | 227092802 | A/G | 0.37 | 0.24 | 0.04 | 9.99×10^-9^ |  | 0.36 | 0.01 | 0.05 | 0.91 |
| rs7903146 | 10 | 114758349 | C/T | 0.71 | 0.23 | 0.03 | 1.11×10^-11^ |  | 0.72 | 0.00 | 0.05 | 0.97 |
| Model was adjusted for pc1 to pc8, study and age. Results were adjusted for age, study, principal components. Chr = chromosome, EAF = effective allele frequency, SE = standard error | | | | | | | | | | | | |
