# Supplementary material for: Genetically Predicted Body Mass Index and Breast Cancer Risk: Mendelian Randomization Analyses of Data from 145,000 Women of European Descent
Source: PLoS Med. 2016 Aug 23;13(8):e1002105. doi: 10.1371/journal.pmed.1002105 (PMC4995025; doi:10.1371/journal.pmed.1002105)
Supplement: S6 Table — (DOCX) [file pmed.1002105.s007.docx]

| **S6 Table. Associations of the weighted BMI genetic score with traditional breast cancer risk factors adjusting for observed BMI (pooled analysis)..** | | | | |
| --- | --- | --- | --- | --- |
| **Outcome** | **Number of participants** | **Summary effect*** | **Standard error** | **p value** |
| **Traditional risk factors**** |  |  |  |  |
| Age (years) | 88,807 | 0.0013 | 0.0034 | 0.70 |
| Age at menarche (years) | 53,990 | -0.0720 | 0.0061 | 3.71×10^-32^ |
| Menopausal status (post vs pre) | 61,686 | 0.0043 | 0.0082 | 0.58 |
| Age at menopause (years) | 26,921 | 0.0315 | 0.0322 | 0.33 |
| Family history of breast cancer (yes vs no) | 47,417 | -0.0106 | 0.0111 | 0.33 |
| Parous (yes vs no) | 62,683 | 0.0121 | 0.0103 | 0.24 |
| Parity (numbers) | 61,837 | 0.0050 | 0.0049 | 0.31 |
| Age at first live birth (years) | 44,735 | -0.0567 | 0.0206 | 0.006 |
| Use of HRT ( Postmenopausal) (ever vs never) | 22,400 | -0.0367 | 0.0128 | 0.004 |
| Breastfeeding (ever vs never) | 43,321 | 0.0125 | 0.0095 | 0.19 |
| Smoking (ever vs never) | 39,562 | 0.0305 | 0.009 | 0.0007 |
| Weight (control) (kg) | 15,410 | 1.3769 | 0.0971 | 2.11×10^-6^ |
| Height (cm) | 50,706 | 0.1964 | 0.0414 | 0.19 |
| *Regression coefficient is presented for continuous variables and natural log-scale odds ratio for dichotomous variables, per unit increase of the genetically predicted BMI. ** The linear regression models fitting weight included only controls, models of all other traditional breast cancer risk factor included all subjects. The total number of subjects is 88,807 (case + control) in our dataset. 22,056 controls have observed BMI. The pre-menopausal controls and the post-menopausal controls do not add up to the total number of control because of missing menopausal status. | | | | |
|  |  |  |  |  |
|  |  |  |  |  |
|  |  |  |  |  |
