# Supplementary material for: Genetically Predicted Body Mass Index and Breast Cancer Risk: Mendelian Randomization Analyses of Data from 145,000 Women of European Descent
Source: PLoS Med. 2016 Aug 23;13(8):e1002105. doi: 10.1371/journal.pmed.1002105 (PMC4995025; doi:10.1371/journal.pmed.1002105)
Supplement: S7 Table — (DOCX) [file pmed.1002105.s008.docx]

| **S7 Table. Association of BMI genetic scores and breast cancer risk, stratified by age group (pooled analysis)..** | | | | |
| --- | --- | --- | --- | --- |
|  | **Case** |  | **BMI genetic scores** | |
| **Age Group** |  | **Control** | **OR (95% CI)** | **p value** |
| Age < 50 | 9627 | 12232 | 0.53(0.38-0.73) | 1.96×10^-5^ |
| Age 50 – 55 | 7554 | 9760 | 0.73(0.52-0.99) | 0.04 |
| Age 55 – 65 | 18473 | 12809 | 0.66(0.51-0.84) | 0.0005 |
| Age > 65 | 10671 | 7681 | 0.85(0.62-1.15) | 0.29 |
| Results are presented for 5 unit BMI genetic scores increase. Model was adjusted for pc1 to pc8, study. | | | | |
|  |  |  |  |  |
