# Supplementary material for: Genetically Predicted Body Mass Index and Breast Cancer Risk: Mendelian Randomization Analyses of Data from 145,000 Women of European Descent
Source: PLoS Med. 2016 Aug 23;13(8):e1002105. doi: 10.1371/journal.pmed.1002105 (PMC4995025; doi:10.1371/journal.pmed.1002105)
Supplement: S8 Table — (DOCX) [file pmed.1002105.s009.docx]

| **S8 Table. Mendelian randomization analysis of observed BMI and breast cancer risk in women using summary data from published BMI GWAS and DRIVE BC GWAS (pooled analysis).** | | | | | | | | | | | | | | | | | | |
| --- | --- | --- | --- | --- | --- | --- | --- | --- | --- | --- | --- | --- | --- | --- | --- | --- | --- | --- |
|  |  |  |  | **SNP and BMI from published GWAS** | | | | |  | **SNP and BC from DRIVE BC GWAS** | | | | | **Instrumental Variable Estimate** | | | |
| **SNP** | **Chr** | **Position** | **Alleles** | **EAF** | **Beta** | **SE** | ***P*** |  | **EAF** | **Beta** | **SE** | **OR (95% CI)** | ***P*** |  | **beta** | **se** | **OR (95% CI)** | ***P*** |
| rs4771122 | 13 | 110154688 | C/T | 0.04 | 0.66 | 0.09 | 7.03×10-14 |  | 0.03 | -0.16 | 0.05 | 0.85(0.75-0.96) | 0.003 |  | -1.20 | 0.43 | 0.59(-0.22-1.39) | 0.006 |
| rs17024393 | 1 | 52361075 | A/T | 0.42 | 0.39 | 0.02 | 4.8×10^-120^ |  | 0.41 | -0.05 | 0.02 | 0.96 (0.92-0.99) | 0.009 |  | -0.58 | 0.23 | 0.52(-0.55-1.58) | 0.01 |
| rs91681123 | 7 | 93197732 | C/G | 0.43 | 0.29 | 0.05 | 2.08×10-10 |  | 0.43 | -0.05 | 0.02 | 0.95(0.92-0.99) | 0.006 |  | -0.85 | 0.34 | 1.87(0.92-2.82) | 0.01 |
| rs3810291 | 19 | 219349752 | C/T | 0.42 | 0.24 | 0.04 | 6.78×10-9 |  | 0.43 | -0.04 | 0.02 | 0.96(0.92-0.99) | 0.01 |  | -0.92 | 0.40 | 0.55(-0.44-1.54) | 0.02 |
| rs1558902 | 16 | 52260843 | A/G | 0.67 | 0.09 | 0.02 | 1.168×10^-12^ |  | 0.68 | -0.06 | 0.02 | 0.95 (0.91-0.99) | 0.008 |  | -3.12 | 1.36 | 7.03(3.82-10.23) | 0.02 |
| rs492400 | 2 | 31129895 | A/G | 0.62 | 0.19 | 0.03 | 8.11×10-10 |  | 0.168 | -0.04 | 0.02 | 0.96(0.92-0.99) | 0.02 |  | -1.09 | 0.50 | 0.54(-0.48-1.56) | 0.03 |
| rs99259168 | 16 | 26918180 | G/A | 0.24 | 0.09 | 0.03 | 9.48×10^-10^ |  | 0.25 | -0.07 | 0.02 | 0.93 (0.89-0.97) | 0.002 |  | -3.89 | 1.80 | 0.55(-0.33-1.43) | 0.03 |
| rs713586 | 2 | 25011512 | C/T | 0.47 | 0.14 | 0.02 | 6.17×10^-22^ |  | 0.48 | -0.04 | 0.02 | 0.96 (0.93-1.00) | 0.03 |  | -1.32 | 0.168 | 0.39(-1.19-1.97) | 0.04 |
| rs7903146 | 10 | 114758349 | C/T | 0.71 | 0.23 | 0.03 | 1.11×10-11 |  | 0.70 | -0.04 | 0.02 | 0.96(0.92-1.00) | 0.04 |  | -0.85 | 0.43 | 0.28(-1.90-2.47) | 0.045 |
| rs543874 | 1 | 176156103 | G/A | 0.19 | 0.22 | 0.03 | 3.56×10^-23^ |  | 0.20 | -0.04 | 0.02 | 0.96 (0.92-1.00) | 0.04 |  | -1.01 | 0.51 | 0.12(-3.85-4.09) | 0.045 |
| rs571312 | 18 | 55990749 | A/C | 0.24 | 0.23 | 0.03 | 6.43×10^-42^ |  | 0.23 | -0.04 | 0.02 | 0.96 (0.92-1.00) | 0.04 |  | -0.88 | 0.45 | 1.70(0.74-2.65) | 0.05 |
| rs12401738 | 1 | 78446761 | A/G | 0.35 | 0.21 | 0.03 | 1.15×10-10 |  | 0.38 | -0.04 | 0.02 | 0.96(0.93-1.00) | 0.05 |  | -0.85 | 0.46 | 0.67(-0.09-1.43) | 0.06 |
| rs987237 | 6 | 72585028 | A/G | 0.61 | 0.13 | 0.02 | 1.61×10^-22^ |  | 0.62 | -0.03 | 0.02 | 0.97 (0.93-1.00) | 0.06 |  | -1.28 | 0.71 | 1.50(0.168-2.36) | 0.07 |
| rs2815752 | 1 | 50911009 | G/A | 0.18 | 0.13 | 0.03 | 2.90×10^-20^ |  | 0.18 | 0.04 | 0.02 | 1.04 (1.00-1.09) | 0.06 |  | 1.65 | 0.94 | 0.63(-0.27-1.54) | 0.08 |
| rs2287019 | 19 | 50894012 | C/T | 0.8 | 0.15 | 0.03 | 1.88×10^-16^ |  | 0.80 | -0.04 | 0.02 | 0.96 (0.92-1.00) | 0.06 |  | -1.32 | 0.76 | 0.62(-0.41-1.168) | 0.08 |
| rs2112347 | 5 | 44877284 | G/A | 0.43 | 0.18 | 0.02 | 3.78×10^-31^ |  | 0.44 | -0.03 | 0.02 | 0.97 (0.94-1.00) | 0.08 |  | -0.85 | 0.49 | 0.72(-0.02-1.45) | 0.08 |
| rs10938397 | 4 | 612827 | C/T | 0.83 | 0.31 | 0.03 | 2.77×10^-49^ |  | 0.83 | -0.04 | 0.02 | 0.96 (0.92-1.01) | 0.08 |  | -0.63 | 0.37 | 1.50(0.60-2.41) | 0.09 |
| rs2867125 | 2 | 75050998 | T/G | 0.63 | 0.1 | 0.02 | 2.17×10^-13^ |  | 0.168 | -0.03 | 0.02 | 0.97 (0.94-1.00) | 0.07 |  | -1.61 | 0.95 | 0.47(-1.16-2.10) | 0.09 |
| rs11057405 | 12 | 122781897 | G/A | 0.90 | 0.31 | 0.06 | 2.02×10-8 |  | 0.90 | 0.05 | 0.03 | 1.05(0.99-1.11) | 0.10 |  | 0.83 | 0.53 | 1.61(0.52-2.69) | 0.11 |
| rs107676168 | 11 | 27682562 | A/T | 0.78 | 0.19 | 0.03 | 4.69×10^-26^ |  | 0.79 | 0.03 | 0.02 | 1.04 (0.99-1.08) | 0.11 |  | 0.91 | 0.58 | 0.168(-0.37-1.65) | 0.12 |
| rs1000940 | 17 | 5283252 | G/A | 0.32 | 0.19 | 0.03 | 1.28×10-8 |  | 0.30 | 0.03 | 0.02 | 1.03(0.99-1.07) | 0.11 |  | 0.80 | 0.52 | 0.69(-0.19-1.56) | 0.12 |
| rs29941 | 19 | 25928179 | C/A | 0.68 | 0.23 | 0.03 | 1.14×10-11 |  | 0.69 | -0.03 | 0.02 | 0.97(0.93-1.01) | 0.13 |  | -0.65 | 0.44 | 0.66(-0.33-1.65) | 0.14 |
| rs10132280 | 14 | 612316862 | C/T | 0.58 | 0.20 | 0.03 | 1.63×10-10 |  | 0.60 | 0.03 | 0.02 | 1.03(0.99-1.06) | 0.15 |  | 0.168 | 0.45 | 1.37(0.51-2.23) | 0.16 |
| rs1808579 | 18 | 21104888 | C/T | 0.53 | 0.17 | 0.03 | 4.17×10-08 |  | 0.53 | -0.03 | 0.02 | 0.98(0.94-1.01) | 0.15 |  | -0.74 | 0.53 | 0.65(-0.39-1.69) | 0.16 |
| rs2365389 | 3 | 39001372 | G/A | 0.67 | 0.06 | 0.02 | 3.01×10^-9^ |  | 0.68 | -0.03 | 0.02 | 0.97 (0.94-1.01) | 0.11 |  | -2.39 | 1.70 | 0.168(-0.41-1.69) | 0.16 |
| rs1514175 | 1 | 103407732 | T/C | 0.07 | 0.19 | 0.04 | 1.50×10^-13^ |  | 0.07 | -0.05 | 0.04 | 0.95 (0.88-1.02) | 0.16 |  | -1.44 | 1.06 | 0.69(-0.37-1.74) | 0.18 |
| rs13107325 | 4 | 51748610 | A/G | 0.45 | 0.18 | 0.03 | 7.41×10-9 |  | 0.48 | -0.02 | 0.02 | 0.98(0.94-1.01) | 0.17 |  | -0.67 | 0.50 | 0.75(-0.08-1.59) | 0.18 |
| rs9374842 | 6 | 120185665 | T/C | 0.74 | 0.23 | 0.04 | 2.67×10-8 |  | 0.78 | -0.03 | 0.02 | 0.97(0.93-1.01) | 0.17 |  | -0.61 | 0.45 | 2.46(-0.11-5.03) | 0.18 |
| rs37316885 | 15 | 747168232 | A/G | 0.43 | 0.07 | 0.02 | 8.16×10^-14^ |  | 0.43 | -0.03 | 0.02 | 0.98 (0.94-1.01) | 0.15 |  | -1.79 | 1.34 | 0.69(-0.34-1.72) | 0.18 |
| rs16907751 | 8 | 213413231 | G/A | 0.72 | 0.22 | 0.03 | 1.17×10-10 |  | 0.73 | -0.03 | 0.02 | 0.97(0.94-1.01) | 0.17 |  | -0.60 | 0.45 | 1.66(0.08-3.24) | 0.18 |
| rs7599312 | 2 | 81375457 | C/T | 0.91 | 0.47 | 0.09 | 3.89×10-8 |  | 0.96 | -0.06 | 0.05 | 0.94(0.84-1.03) | 0.17 |  | -0.69 | 0.52 | 1.48(0.28-2.68) | 0.19 |
| rs17001654 | 4 | 77129568 | G/C | 0.15 | 0.31 | 0.05 | 7.76×10-9 |  | 0.15 | -0.03 | 0.02 | 0.97(0.92-1.02) | 0.18 |  | -0.53 | 0.41 | 0.49(-1.96-2.93) | 0.19 |
| rs13191362 | 6 | 163033350 | A/G | 0.88 | 0.28 | 0.05 | 7.34×10-9 |  | 0.87 | 0.04 | 0.03 | 1.04(0.98-1.09) | 0.18 |  | 0.63 | 0.48 | 0.75(-0.21-1.71) | 0.20 |
| rs3817334 | 11 | 40291740 | C/T | 0.60 | 0.17 | 0.03 | 1.61×10-8 |  | 0.168 | -0.02 | 0.02 | 0.98(0.94-1.01) | 0.21 |  | -0.66 | 0.54 | 0.82(0.05-1.59) | 0.22 |
| rs2836754 | 21 | 47607569 | T/C | 0.41 | 0.06 | 0.02 | 1.59×10^-12^ |  | 0.41 | 0.02 | 0.02 | 1.02 (0.99-1.06) | 0.19 |  | 1.95 | 1.63 | 0.77(-0.18-1.73) | 0.23 |
| rs1528435 | 2 | 181550962 | T/C | 0.63 | 0.18 | 0.03 | 1.20×10-8 |  | 0.63 | -0.02 | 0.02 | 0.98(0.94-1.01) | 0.22 |  | -0.60 | 0.51 | 1.44(-0.01-2.89) | 0.23 |
| rs2176598 | 11 | 438168278 | T/C | 0.25 | 0.20 | 0.04 | 2.97×10-8 |  | 0.25 | -0.02 | 0.02 | 0.98(0.94-1.02) | 0.23 |  | -0.61 | 0.52 | 1.25(0.29-2.22) | 0.24 |
| rs2241423 | 15 | 65873892 | G/A | 0.78 | 0.13 | 0.02 | 1.19×10^-18^ |  | 0.78 | -0.02 | 0.02 | 0.98 (0.94-1.02) | 0.23 |  | -0.95 | 0.81 | 0.66(-1.18-2.50) | 0.24 |
| rs13078807 | 3 | 85966840 | G/A | 0.2 | 0.1 | 0.02 | 3.94×10^-11^ |  | 0.19 | -0.03 | 0.02 | 0.98 (0.93-1.02) | 0.24 |  | -1.27 | 1.11 | 1.76(-1.06-4.58) | 0.26 |
| rs206936 | 6 | 81792112 | A/C | 0.36 | 0.19 | 0.03 | 2.60×10-8 |  | 0.34 | 0.02 | 0.02 | 1.02(0.98-1.06) | 0.27 |  | 0.53 | 0.49 | 1.16(0.26-2.06) | 0.28 |
| rs3849570 | 3 | 34410847 | G/A | 0.21 | 0.06 | 0.02 | 3.02×10^-8^ |  | 0.20 | -0.03 | 0.02 | 0.97 (0.93-1.02) | 0.27 |  | -2.12 | 2.03 | 0.84(-0.16-1.84) | 0.30 |
| rs16851483 | 3 | 141275436 | T/G | 0.07 | 0.48 | 0.08 | 3.55×10-10 |  | 0.07 | -0.04 | 0.04 | 0.96(0.89-1.03) | 0.30 |  | -0.40 | 0.39 | 0.87(0.01-1.73) | 0.31 |
| rs1928295 | 9 | 120378483 | T/C | 0.55 | 0.19 | 0.03 | 7.91×10-10 |  | 0.57 | -0.02 | 0.02 | 0.98(0.95-1.02) | 0.32 |  | -0.46 | 0.46 | 1.14(0.28-1.99) | 0.32 |
| rs4787491 | 16 | 30015337 | G/A | 0.51 | 0.22 | 0.04 | 2.70×10-8 |  | 0.54 | 0.02 | 0.02 | 1.02(0.98-1.06) | 0.35 |  | 0.41 | 0.44 | 0.75(-2.00-3.49) | 0.36 |
| rs977747 | 1 | 47684677 | T/G | 0.40 | 0.17 | 0.03 | 2.18×10-8 |  | 0.42 | -0.02 | 0.02 | 0.98(0.95-1.02) | 0.35 |  | -0.48 | 0.52 | 0.92(-0.02-1.86) | 0.36 |
| rs10150332 | 14 | 79006717 | C/T | 0.21 | 0.13 | 0.03 | 2.75×10^-11^ |  | 0.21 | -0.02 | 0.02 | 0.98 (0.94-1.02) | 0.35 |  | -0.75 | 0.83 | 1.09(0.10-2.07) | 0.36 |
| rs2176040 | 2 | 227092802 | A/G | 0.37 | 0.24 | 0.04 | 9.99×10-9 |  | 0.35 | -0.02 | 0.02 | 0.98(0.95-1.02) | 0.37 |  | -0.33 | 0.38 | 0.93(-0.12-1.99) | 0.37 |
| rs758747 | 16 | 3627358 | T/C | 0.27 | 0.23 | 0.04 | 7.47×10-10 |  | 0.27 | 0.02 | 0.02 | 1.02(0.98-1.06) | 0.37 |  | 0.41 | 0.46 | 0.94(-0.11-1.98) | 0.38 |
| rs17724992 | 19 | 153537893 | G/T | 0.42 | 0.17 | 0.03 | 8.85×10-9 |  | 0.43 | -0.02 | 0.02 | 0.99(0.95-1.02) | 0.39 |  | -0.44 | 0.52 | 1.08(-0.72-2.88) | 0.39 |
| rs7715256 | 5 | 18454825 | A/G | 0.75 | 0.19 | 0.04 | 3.42×10-8 |  | 0.74 | 0.02 | 0.02 | 1.02(0.98-1.06) | 0.38 |  | 0.47 | 0.55 | 1.06(-0.36-2.49) | 0.39 |
| rs17094222 | 10 | 102395440 | C/T | 0.21 | 0.25 | 0.04 | 5.94×10-11 |  | 0.21 | -0.02 | 0.02 | 0.98(0.94-1.02) | 0.39 |  | -0.38 | 0.45 | 0.96(-0.08-2.01) | 0.40 |
| rs16877694 | 9 | 111932342 | C/T | 0.37 | 0.17 | 0.03 | 2.67×10-8 |  | 0.35 | -0.02 | 0.02 | 0.98(0.95-1.02) | 0.40 |  | -0.45 | 0.54 | 1.06(-1.78-3.90) | 0.40 |
| rs11583200 | 1 | 50559820 | C/T | 0.40 | 0.18 | 0.03 | 1.48×10-8 |  | 0.38 | -0.01 | 0.02 | 0.99(0.95-1.02) | 0.41 |  | -0.41 | 0.50 | 0.98(0.03-1.94) | 0.42 |
| rs10733682 | 9 | 129460914 | A/G | 0.48 | 0.17 | 0.03 | 1.83×10-8 |  | 0.47 | -0.01 | 0.02 | 0.99(0.95-1.02) | 0.41 |  | -0.43 | 0.53 | 1.00(0.25-1.75) | 0.42 |
| rs12885454 | 14 | 29736838 | C/A | 0.168 | 0.21 | 0.03 | 1.94×10-10 |  | 0.168 | 0.01 | 0.02 | 1.01(0.98-1.05) | 0.47 |  | 0.32 | 0.44 | 1.37(0.51-2.23) | 0.47 |
| rs7243357 | 18 | 56883319 | T/G | 0.81 | 0.22 | 0.04 | 3.86×10-8 |  | 0.83 | -0.02 | 0.02 | 0.98(0.94-1.03) | 0.48 |  | -0.37 | 0.53 | 0.69(-0.34-1.72) | 0.48 |
| rs9914578 | 17 | 2005136 | G/C | 0.23 | 0.20 | 0.04 | 2.07×10-8 |  | 0.20 | -0.02 | 0.02 | 0.99(0.94-1.03) | 0.48 |  | -0.38 | 0.54 | 0.69(-0.37-1.74) | 0.48 |
| rs2890652 | 2 | 1426716801 | C/T | 0.18 | 0.09 | 0.03 | 1.35×10^-10^ |  | 0.17 | 0.02 | 0.02 | 1.02 (0.97-1.06) | 0.48 |  | 0.90 | 1.31 | 2.46(-0.11-5.03) | 0.49 |
| rs17405819 | 8 | 76806584 | T/C | 0.70 | 0.22 | 0.03 | 2.07×10-11 |  | 0.69 | -0.01 | 0.02 | 0.99(0.95-1.02) | 0.50 |  | -0.28 | 0.43 | 0.75(-0.08-1.59) | 0.51 |
| rs7359397 | 16 | 28793160 | T/C | 0.4 | 0.15 | 0.02 | 1.88×10^-20^ |  | 0.37 | 0.01 | 0.02 | 1.01 (0.98-1.05) | 0.52 |  | 0.39 | 0.61 | 1.48(0.28-2.68) | 0.52 |
| rs9816226 | 3 | 187317193 | T/A | 0.82 | 0.14 | 0.03 | 1.69×10^-18^ |  | 0.83 | 0.01 | 0.02 | 1.01 (0.97-1.06) | 0.53 |  | 0.51 | 0.81 | 1.66(0.08-3.24) | 0.53 |
| rs4836133 | 5 | 104869038 | C/T | 0.09 | 0.31 | 0.05 | 8.45×10-9 |  | 0.08 | -0.02 | 0.03 | 0.98(0.92-1.04) | 0.56 |  | -0.29 | 0.49 | 0.75(-0.21-1.71) | 0.56 |
| rs11191560 | 10 | 124360002 | A/C | 0.48 | 0.07 | 0.02 | 1.97×10^-9^ |  | 0.53 | -0.01 | 0.02 | 0.99 (0.96-1.02) | 0.56 |  | -0.72 | 1.25 | 0.49(-1.96-2.93) | 0.56 |
| rs71168727 | 15 | 73093991 | T/C | 0.67 | 0.19 | 0.03 | 3.92×10-9 |  | 0.67 | -0.01 | 0.02 | 0.99(0.95-1.03) | 0.60 |  | -0.26 | 0.49 | 0.77(-0.18-1.73) | 0.60 |
| rs657452 | 1 | 49589847 | A/G | 0.39 | 0.23 | 0.03 | 5.48×10-13 |  | 0.39 | -0.01 | 0.02 | 0.99(0.96-1.03) | 0.61 |  | -0.20 | 0.39 | 0.82(0.05-1.59) | 0.62 |
| rs12444979 | 16 | 19841101 | C/T | 0.87 | 0.17 | 0.03 | 2.91×10^-21^ |  | 0.86 | 0.01 | 0.03 | 1.01 (0.96-1.06) | 0.62 |  | 0.36 | 0.74 | 1.44(-0.01-2.89) | 0.62 |
| rs9400239 | 6 | 108977663 | C/T | 0.69 | 0.19 | 0.03 | 1.61×10-8 |  | 0.71 | 0.01 | 0.02 | 1.01(0.97-1.05) | 0.168 |  | 0.23 | 0.49 | 1.25(0.29-2.22) | 0.65 |
| rs887912 | 2 | 59156381 | T/C | 0.29 | 0.1 | 0.02 | 1.79×10^-12^ |  | 0.30 | -0.01 | 0.02 | 0.99 (0.96-1.03) | 0.66 |  | -0.41 | 0.94 | 0.66(-1.18-2.50) | 0.66 |
| rs4929949 | 11 | 8561169 | C/T | 0.52 | 0.06 | 0.02 | 2.80×10^-9^ |  | 0.52 | 0.01 | 0.02 | 1.01 (0.97-1.04) | 0.69 |  | 0.57 | 1.44 | 1.76(-1.06-4.58) | 0.69 |
| rs11688816 | 2 | 63053048 | G/A | 0.53 | 0.17 | 0.03 | 1.89×10-8 |  | 0.54 | -0.01 | 0.02 | 0.99(0.96-1.03) | 0.74 |  | -0.17 | 0.51 | 0.84(-0.16-1.84) | 0.74 |
| rs1167827 | 7 | 75163169 | G/A | 0.55 | 0.20 | 0.03 | 6.33×10-10 |  | 0.58 | 0.01 | 0.02 | 1.01(0.97-1.04) | 0.75 |  | 0.15 | 0.46 | 1.16(0.26-2.06) | 0.75 |
| rs205262 | 6 | 345631168 | G/A | 0.27 | 0.22 | 0.04 | 1.75×10-10 |  | 0.27 | -0.01 | 0.02 | 0.99(0.96-1.03) | 0.75 |  | -0.14 | 0.44 | 0.87(0.01-1.73) | 0.75 |
| rs2820292 | 1 | 201784287 | C/A | 0.56 | 0.20 | 0.03 | 1.83×10-10 |  | 0.55 | 0.01 | 0.02 | 1.01(0.97-1.04) | 0.77 |  | 0.13 | 0.43 | 1.14(0.28-1.99) | 0.77 |
| rs11847697 | 14 | 29584863 | T/C | 0.04 | 0.17 | 0.05 | 5.76×10^-11^ |  | 0.04 | -0.01 | 0.05 | 0.99 (0.90-1.09) | 0.84 |  | -0.29 | 1.40 | 0.75(-2.00-3.49) | 0.84 |
| rs4740619 | 9 | 15634326 | T/C | 0.54 | 0.18 | 0.03 | 4.56×10-9 |  | 0.56 | 0.00 | 0.02 | 1.00(0.96-1.03) | 0.86 |  | -0.08 | 0.48 | 0.92(-0.02-1.86) | 0.86 |
| rs7899106 | 10 | 87410904 | G/A | 0.05 | 0.40 | 0.07 | 2.96×10-8 |  | 0.05 | 0.01 | 0.04 | 1.01(0.93-1.09) | 0.87 |  | 0.08 | 0.50 | 1.09(0.10-2.07) | 0.87 |
| rs2080454 | 16 | 49062590 | C/A | 0.41 | 0.17 | 0.03 | 8.60×10-9 |  | 0.38 | 0.00 | 0.02 | 1.00(0.96-1.03) | 0.90 |  | -0.07 | 0.54 | 0.93(-0.12-1.99) | 0.90 |
| rs17203016 | 2 | 208255518 | G/A | 0.20 | 0.21 | 0.04 | 3.41×10-8 |  | 0.20 | 0.00 | 0.02 | 1.00(0.95-1.04) | 0.91 |  | -0.06 | 0.53 | 0.94(-0.11-1.98) | 0.91 |
| rs7138803 | 12 | 48533735 | A/G | 0.38 | 0.12 | 0.02 | 1.82×10^-17^ |  | 0.38 | 0.00 | 0.02 | 1.00 (0.97-1.04) | 0.93 |  | 0.06 | 0.73 | 1.06(-0.36-2.49) | 0.93 |
| rs10968576 | 9 | 28404339 | G/A | 0.31 | 0.11 | 0.02 | 2.65×10^-13^ |  | 0.32 | 0.00 | 0.02 | 1.00 (0.96-1.04) | 0.94 |  | 0.07 | 0.92 | 1.08(-0.72-2.88) | 0.94 |
| rs14412168 | 13 | 79580919 | A/G | 0.61 | 0.17 | 0.03 | 2.96×10-8 |  | 0.60 | 0.00 | 0.02 | 1.00(0.96-1.03) | 0.95 |  | -0.04 | 0.53 | 0.96(-0.08-2.01) | 0.95 |
| rs1555543 | 1 | 96717385 | C/A | 0.59 | 0.06 | 0.02 | 3.68×10^-10^ |  | 0.58 | 0.00 | 0.02 | 1.00 (0.97-1.04) | 0.97 |  | 0.06 | 1.45 | 1.06(-1.78-3.90) | 0.97 |
| rs2075650 | 19 | 45395619 | A/G | 0.85 | 0.26 | 0.05 | 1.25×10-8 |  | 0.85 | 0.00 | 0.03 | 1.00(0.95-1.05) | 0.97 |  | -0.02 | 0.49 | 0.98(0.03-1.94) | 0.97 |
| rs7239883 | 18 | 40147671 | G/A | 0.39 | 0.23 | 0.04 | 1.51×10-8 |  | 0.38 | 0.00 | 0.02 | 1.00(0.97-1.03) | 1.00 |  | 0.00 | 0.38 | 1.00(0.25-1.75) | 1.00 |

Published GWAS results were presented in 1kg/m^2^ unit increase. Instrumental variable results are presented for per 5kg/m^2^ increase of SNP. The beta of instrumental variables was computed using $\beta_{YX}=5\times\frac{\beta_{YG}}{\beta_{XG}}$. The standard error for instrument variable was computed using $SE_{YX}=\sqrt{\left(\left(\frac{S_{YG}}{\beta_{XG}}\right)^{2}+\frac{\left(S_{XG}\beta_{YG}\right)^{2}}{\beta_{XG}^{4}}\right)}$

Chr = chromosome, EAF = effective allele frequency, SE = standard error BMI results from published GWAS are presented for 1 unit kg/m2. Instrumental variable estimates are presented for 5 kg/m^2^
