# Supplementary material for: Genetically Predicted Body Mass Index and Breast Cancer Risk: Mendelian Randomization Analyses of Data from 145,000 Women of European Descent
Source: PLoS Med. 2016 Aug 23;13(8):e1002105. doi: 10.1371/journal.pmed.1002105 (PMC4995025; doi:10.1371/journal.pmed.1002105)
Supplement: S9 Table — (DOCX) [file pmed.1002105.s010.docx]

| **S9 Table. Associations of the 84 SNPs with breast cancer risk in the Breast Cancer Association Consortium (pooled analysis).** | | | | | | | | | | | |
| --- | --- | --- | --- | --- | --- | --- | --- | --- | --- | --- | --- |
|  |  |  |  | **All women combined N=88807** | |  | **Premenopausal women N=13231** | |  | **Postmenopausal women N=31480** | |
| **SNP** | **Chr** | **Position** | **Alleles** | **OR (95% CI)** | ***P*** |  | **OR (95% CI)** | ***P*** |  | **OR (95% CI)** | ***P*** |
| rs12401738 | 1 | 78446761 | A/G | 0.98(0.96-1.00) | 0.05 |  | 1.02(0.96-1.08) | 0.48 |  | 0.93(0.89-0.96) | 5.72×10^-5^ |
| rs1558902 | 16 | 53803574 | A/T | 0.93(0.91-0.95) | 2.77×10^-14^ |  | 0.92(0.87-0.97) | 0.0019 |  | 0.94(0.91-0.98) | 0.0006 |
| rs713586 | 2 | 25158008 | C/T | 0.94(0.92-0.97) | 1.82×10^-6^ |  | 0.93(0.87-1.00) | 0.04 |  | 0.94(0.90-0.98) | 0.003 |
| rs10150332 | 14 | 799369168 | C/T | 1.02(1.00-1.04) | 0.12 |  | 0.96(0.90-1.02) | 0.19 |  | 1.05(1.01-1.09) | 0.02 |
| rs7903146 | 10 | 114758349 | C/T | 0.96(0.94-0.98) | 7.01×10^-5^ |  | 0.93(0.88-0.99) | 0.02 |  | 0.96(0.92-1.00) | 0.03 |
| rs17024393 | 1 | 110154688 | C/T | 0.93(0.87-0.98) | 0.007 |  | 0.86(0.71-1.01) | 0.04 |  | 0.9(0.81-0.99) | 0.03 |
| rs4771122 | 13 | 28020180 | G/A | 0.98(0.96-1.00) | 0.06 |  | 1.00(0.94-1.06) | 1.00 |  | 0.96(0.92-1.00) | 0.03 |
| rs107676168 | 11 | 27725986 | A/T | 0.99(0.97-1.02) | 0.54 |  | 0.99(0.92-1.05) | 0.65 |  | 0.96(0.92-1.00) | 0.07 |
| rs3810291 | 19 | 47569003 | A/G | 0.98(0.95-1.00) | 0.01 |  | 1.00(0.95-1.06) | 0.92 |  | 0.97(0.94-1.00) | 0.08 |
| rs2867125 | 2 | 622827 | C/T | 0.96(0.94-0.99) | 0.003 |  | 1.02(0.96-1.09) | 0.51 |  | 0.96(0.92-1.01) | 0.09 |
| rs11688816 | 2 | 63053048 | G/A | 1.01(0.99-1.03) | 0.49 |  | 1.05(1.00-1.11) | 0.04 |  | 1.03(0.99-1.06) | 0.13 |
| rs11847697 | 14 | 30515112 | T/C | 0.96(0.90-1.03) | 0.24 |  | 1.00(0.83-1.17) | 0.99 |  | 0.92(0.82-1.03) | 0.14 |
| rs7239883 | 18 | 40147671 | G/A | 1.00(0.98-1.02) | 0.89 |  | 1.00(0.94-1.06) | 1.00 |  | 0.97(0.94-1.01) | 0.15 |
| rs1808579 | 18 | 21104888 | C/T | 1.01(0.99-1.03) | 0.40 |  | 0.96(0.91-1.02) | 0.17 |  | 1.02(0.99-1.06) | 0.17 |
| rs2080454 | 16 | 49062590 | C/A | 1.00(0.98-1.02) | 0.95 |  | 1.06(1.00-1.12) | 0.04 |  | 0.97(0.94-1.01) | 0.17 |
| rs10733682 | 9 | 129460914 | A/G | 0.97(0.95-0.99) | 0.009 |  | 0.98(0.93-1.04) | 0.57 |  | 0.98(0.94-1.01) | 0.18 |
| rs12444979 | 16 | 19933600 | C/T | 0.99(0.96-1.02) | 0.49 |  | 0.98(0.91-1.05) | 0.57 |  | 0.97(0.92-1.02) | 0.19 |
| rs571312 | 18 | 57839769 | A/C | 0.97(0.95-1.00) | 0.02 |  | 0.95(0.89-1.01) | 0.12 |  | 0.98(0.94-1.01) | 0.20 |
| rs987237 | 6 | 50803050 | G/A | 1.01(0.98-1.03) | 0.52 |  | 1.04(0.97-1.11) | 0.23 |  | 0.97(0.93-1.02) | 0.23 |
| rs1528435 | 2 | 181550962 | T/C | 0.97(0.95-0.99) | 0.01 |  | 0.97(0.91-1.02) | 0.22 |  | 0.98(0.94-1.02) | 0.27 |
| rs29941 | 19 | 34309532 | G/A | 0.99(0.97-1.01) | 0.54 |  | 1.01(0.96-1.06) | 0.69 |  | 0.98(0.95-1.02) | 0.27 |
| rs7243357 | 18 | 56883319 | T/G | 1.00(0.97-1.02) | 0.92 |  | 0.98(0.92-1.05) | 0.66 |  | 0.98(0.93-1.02) | 0.30 |
| rs206936 | 6 | 34302869 | G/A | 0.99(0.97-1.01) | 0.43 |  | 1.04(0.97-1.10) | 0.29 |  | 0.98(0.94-1.02) | 0.30 |
| rs2176040 | 2 | 227092802 | A/G | 1.00(0.98-1.02) | 0.92 |  | 1.01(0.96-1.07) | 0.62 |  | 0.98(0.95-1.02) | 0.31 |
| rs1928295 | 9 | 120378483 | T/C | 1.00(0.98-1.02) | 0.85 |  | 0.99(0.93-1.04) | 0.62 |  | 0.98(0.95-1.02) | 0.31 |
| rs99259168 | 16 | 31129895 | A/G | 1.00(0.98-1.02) | 0.91 |  | 0.95(0.90-1.01) | 0.07 |  | 1.02(0.98-1.05) | 0.32 |
| rs10938397 | 4 | 45182527 | G/A | 0.99(0.97-1.01) | 0.47 |  | 0.96(0.91-1.01) | 0.16 |  | 0.98(0.95-1.02) | 0.34 |
| rs17001654 | 4 | 77129568 | G/C | 0.98(0.95-1.01) | 0.19 |  | 0.94(0.86-1.02) | 0.14 |  | 0.98(0.92-1.03) | 0.34 |
| rs17203016 | 2 | 208255518 | G/A | 1.02(0.99-1.04) | 0.24 |  | 1.02(0.96-1.09) | 0.49 |  | 1.02(0.98-1.06) | 0.34 |
| rs2075650 | 19 | 45395619 | A/G | 1.02(0.99-1.05) | 0.23 |  | 1.00(0.92-1.09) | 0.92 |  | 1.02(0.97-1.08) | 0.37 |
| rs1555543 | 1 | 96944797 | C/A | 1.00(0.97-1.02) | 0.81 |  | 0.96(0.90-1.03) | 0.24 |  | 1.02(0.98-1.06) | 0.38 |
| rs9914578 | 17 | 2005136 | G/C | 1.02(1.00-1.04) | 0.09 |  | 1.01(0.95-1.07) | 0.74 |  | 1.02(0.98-1.06) | 0.38 |
| rs758747 | 16 | 3627358 | T/C | 1.00(0.97-1.02) | 0.68 |  | 0.99(0.94-1.05) | 0.81 |  | 0.98(0.95-1.02) | 0.39 |
| rs2836754 | 21 | 40291740 | C/T | 1.00(0.98-1.02) | 0.71 |  | 1.01(0.96-1.07) | 0.66 |  | 1.01(0.98-1.05) | 0.42 |
| rs2112347 | 5 | 75015242 | T/G | 0.98(0.96-1.00) | 0.03 |  | 0.98(0.93-1.04) | 0.52 |  | 0.99(0.95-1.02) | 0.43 |
| rs9400239 | 6 | 108977663 | C/T | 1.00(0.97-1.02) | 0.67 |  | 0.98(0.93-1.04) | 0.55 |  | 1.01(0.98-1.05) | 0.43 |
| rs17094222 | 10 | 102395440 | C/T | 1.01(0.98-1.04) | 0.54 |  | 1.05(0.98-1.12) | 0.18 |  | 1.02(0.97-1.06) | 0.44 |
| rs9816226 | 3 | 185834499 | T/A | 1.01(0.98-1.04) | 0.41 |  | 0.99(0.92-1.06) | 0.83 |  | 1.02(0.97-1.06) | 0.44 |
| rs7138803 | 12 | 50247468 | A/G | 0.99(0.97-1.01) | 0.19 |  | 0.93(0.87-0.98) | 0.002 |  | 0.99(0.95-1.02) | 0.46 |
| rs2241423 | 15 | 68086838 | G/A | 1.00(0.98-1.03) | 0.69 |  | 1.00(0.94-1.06) | 0.96 |  | 1.01(0.98-1.05) | 0.46 |
| rs16877694 | 9 | 111932342 | C/T | 1.00(0.98-1.02) | 0.79 |  | 1.00(0.94-1.05) | 0.86 |  | 1.01(0.98-1.05) | 0.46 |
| rs887912 | 2 | 59302877 | T/C | 1.00(0.98-1.02) | 0.78 |  | 0.95(0.90-1.01) | 0.11 |  | 0.99(0.95-1.02) | 0.47 |
| rs2287019 | 19 | 46202172 | C/T | 0.96(0.93-0.99) | 0.009 |  | 0.94(0.86-1.01) | 0.09 |  | 0.98(0.94-1.03) | 0.48 |
| rs3817334 | 11 | 47650993 | T/C | 1.01(0.98-1.03) | 0.51 |  | 1.05(0.99-1.11) | 0.13 |  | 1.01(0.97-1.05) | 0.51 |
| rs11583200 | 1 | 50559820 | C/T | 0.98(0.96-1.00) | 0.11 |  | 0.96(0.91-1.02) | 0.20 |  | 0.99(0.95-1.02) | 0.52 |
| rs16851483 | 3 | 141275436 | T/G | 1.03(0.99-1.07) | 0.15 |  | 1.05(0.95-1.16) | 0.33 |  | 1.02(0.96-1.09) | 0.53 |
| rs7599312 | 2 | 213413231 | G/A | 0.96(0.94-0.98) | 0.0004 |  | 0.94(0.88-1.00) | 0.06 |  | 0.99(0.95-1.03) | 0.53 |
| rs91681123 | 7 | 93197732 | C/G | 1.00(0.98-1.02) | 0.96 |  | 0.95(0.90-1.00) | 0.05 |  | 0.99(0.96-1.02) | 0.54 |
| rs4787491 | 16 | 30015337 | G/A | 1.00(0.98-1.02) | 0.90 |  | 0.98(0.92-1.03) | 0.36 |  | 0.99(0.96-1.02) | 0.56 |
| rs17724992 | 19 | 18454825 | A/G | 1.01(0.98-1.03) | 0.56 |  | 0.95(0.89-1.00) | 0.07 |  | 1.01(0.97-1.05) | 0.58 |
| rs977747 | 1 | 47684677 | T/G | 1.00(0.98-1.02) | 0.81 |  | 1.00(0.95-1.06) | 0.86 |  | 1.01(0.98-1.04) | 0.59 |
| rs13107325 | 4 | 103188709 | T/C | 1.00(0.96-1.04) | 0.93 |  | 1.06(0.95-1.17) | 0.32 |  | 0.98(0.91-1.05) | 0.60 |
| rs1514175 | 1 | 749911684 | A/G | 1.00(0.98-1.02) | 1.00 |  | 0.95(0.90-1.01) | 0.09 |  | 1.01(0.97-1.04) | 0.61 |
| rs10968576 | 9 | 28414339 | G/A | 1.00(0.98-1.02) | 0.93 |  | 0.98(0.93-1.04) | 0.53 |  | 1.01(0.97-1.04) | 0.62 |
| rs71168727 | 15 | 73093991 | T/C | 0.99(0.97-1.01) | 0.51 |  | 0.97(0.91-1.03) | 0.28 |  | 0.99(0.96-1.03) | 0.17 |
| rs12885454 | 14 | 29736838 | C/A | 1.01(0.99-1.03) | 0.50 |  | 1.02(0.97-1.07) | 0.44 |  | 1.01(0.97-1.04) | 0.17 |
| rs10132280 | 14 | 25928179 | C/A | 1.00(0.98-1.03) | 0.75 |  | 1.04(0.98-1.10) | 0.19 |  | 0.99(0.95-1.03) | 0.65 |
| rs543874 | 1 | 177889480 | G/A | 0.97(0.95-1.00) | 0.04 |  | 0.97(0.90-1.03) | 0.33 |  | 0.99(0.95-1.03) | 0.65 |
| rs657452 | 1 | 49589847 | A/G | 0.98(0.96-1.00) | 0.10 |  | 0.96(0.90-1.01) | 0.10 |  | 0.99(0.96-1.03) | 0.66 |
| rs14412168 | 13 | 79580919 | A/G | 0.99(0.96-1.01) | 0.39 |  | 0.99(0.92-1.06) | 0.71 |  | 1.01(0.97-1.05) | 0.67 |
| rs13078807 | 3 | 85884150 | G/A | 0.99(0.97-1.02) | 0.67 |  | 0.98(0.91-1.05) | 0.59 |  | 0.99(0.95-1.03) | 0.69 |
| rs1167827 | 7 | 75163169 | G/A | 1.01(0.99-1.03) | 0.30 |  | 1.01(0.96-1.06) | 0.66 |  | 0.99(0.96-1.03) | 0.70 |
| rs2365389 | 3 | 612316862 | C/T | 0.99(0.97-1.01) | 0.53 |  | 0.97(0.92-1.03) | 0.30 |  | 1.01(0.97-1.04) | 0.72 |
| rs1000940 | 17 | 5283252 | G/A | 1.01(0.99-1.03) | 0.30 |  | 0.98(0.92-1.03) | 0.42 |  | 1.01(0.97-1.04) | 0.73 |
| rs2890652 | 2 | 142959931 | C/T | 1.00(0.97-1.02) | 0.77 |  | 0.99(0.91-1.06) | 0.69 |  | 1.01(0.96-1.05) | 0.76 |
| rs17405819 | 8 | 76806584 | T/C | 0.97(0.95-1.00) | 0.02 |  | 1.00(0.94-1.05) | 0.87 |  | 0.99(0.96-1.03) | 0.77 |
| rs3849570 | 3 | 81792112 | A/C | 1.00(0.98-1.03) | 0.78 |  | 0.99(0.93-1.05) | 0.79 |  | 0.99(0.96-1.03) | 0.77 |
| rs11057405 | 12 | 122781897 | G/A | 1.02(0.99-1.06) | 0.21 |  | 1.01(0.91-1.11) | 0.90 |  | 0.99(0.93-1.06) | 0.80 |
| rs4740619 | 9 | 15634326 | T/C | 1.01(0.99-1.03) | 0.50 |  | 0.98(0.93-1.03) | 0.51 |  | 1.00(0.97-1.04) | 0.80 |
| rs7715256 | 5 | 153537893 | G/T | 0.99(0.97-1.01) | 0.53 |  | 0.96(0.91-1.01) | 0.16 |  | 1.00(0.97-1.04) | 0.80 |
| rs492400 | 2 | 219349752 | C/T | 1.02(0.99-1.04) | 0.15 |  | 1.02(0.96-1.07) | 0.50 |  | 1.00(0.97-1.04) | 0.81 |
| rs7899106 | 10 | 87410904 | G/A | 1.00(0.95-1.05) | 0.99 |  | 1.05(0.93-1.17) | 0.39 |  | 1.01(0.93-1.09) | 0.81 |
| rs2820292 | 1 | 201784287 | C/A | 1.00(0.98-1.02) | 0.85 |  | 0.97(0.92-1.02) | 0.25 |  | 1.00(0.96-1.03) | 0.82 |
| rs2176598 | 11 | 438168278 | T/C | 1.00(0.97-1.02) | 0.68 |  | 1.04(0.98-1.10) | 0.17 |  | 1.00(0.96-1.03) | 0.83 |
| rs7359397 | 16 | 28885659 | T/C | 1.01(0.99-1.03) | 0.55 |  | 1.02(0.96-1.07) | 0.55 |  | 1.00(0.96-1.03) | 0.84 |
| rs4929949 | 11 | 8604593 | C/T | 1.00(0.98-1.02) | 0.90 |  | 1.01(0.96-1.07) | 0.63 |  | 1.00(0.96-1.03) | 0.88 |
| rs205262 | 6 | 345631168 | G/A | 1.01(0.99-1.03) | 0.31 |  | 0.98(0.93-1.04) | 0.60 |  | 1.00(0.96-1.03) | 0.88 |
| rs13191362 | 6 | 163033350 | A/G | 1.03(1.00-1.06) | 0.047 |  | 0.96(0.87-1.04) | 0.32 |  | 1.00(0.95-1.06) | 0.90 |
| rs2815752 | 1 | 72812440 | A/G | 0.99(0.97-1.01) | 0.22 |  | 0.98(0.93-1.03) | 0.49 |  | 1.00(0.97-1.03) | 0.91 |
| rs4836133 | 5 | 124332103 | A/C | 0.99(0.98-1.01) | 0.59 |  | 0.96(0.91-1.02) | 0.17 |  | 1.00(0.97-1.03) | 0.95 |
| rs9374842 | 6 | 120185665 | T/C | 1.01(0.99-1.04) | 0.27 |  | 0.98(0.92-1.05) | 0.58 |  | 1.00(0.96-1.04) | 0.97 |
| rs37316885 | 15 | 51748610 | A/G | 0.98(0.96-1.00) | 0.12 |  | 0.97(0.92-1.03) | 0.29 |  | 1.00(0.97-1.03) | 0.98 |
| rs11191560 | 10 | 104869038 | C/T | 0.98(0.94-1.01) | 0.20 |  | 0.95(0.85-1.05) | 0.31 |  | 1.00(0.94-1.06) | 0.98 |
| rs16907751 | 8 | 81375457 | C/T | 0.99(0.96-1.03) | 0.75 |  | 1.01(0.91-1.10) | 0.87 |  | 1.00(0.94-1.06) | 0.99 |

Chr = chromosome, EAF = effective allele frequency, SE = standard error
