# Supplementary material for: Genetically Predicted Body Mass Index and Breast Cancer Risk: Mendelian Randomization Analyses of Data from 145,000 Women of European Descent
Source: PLoS Med. 2016 Aug 23;13(8):e1002105. doi: 10.1371/journal.pmed.1002105 (PMC4995025; doi:10.1371/journal.pmed.1002105)
Supplement: S10 Table — (DOCX) [file pmed.1002105.s011.docx]

| **S10 Table: Association of breast cancer risk with 84 BMI-related SNPs (pooled analysis).** | | | | | | | | | | | | | | |
| --- | --- | --- | --- | --- | --- | --- | --- | --- | --- | --- | --- | --- | --- | --- |
|  |  |  |  |  | **BCAC** | | |  | **GAME-ON DRIVE** | | |  | **Combined** | |
| **SNP** | **Chr** | **Position** | **Gene** | **Alleles** | **EAF** | **OR (95% CI)** | ***P*** |  | **EAF** | **OR (95% CI)** | ***P*** |  | **OR (95% CI)*** | ***P*** |
| rs7599312 | 2 | 213413231 | LMX1B(B,N) | G/A | 0.72 | 0.96(0.94-0.98) | 0.0004 |  | 0.96 | 0.94(0.84-1.03) | 0.17 |  | 0.96(0.94-0.98) | 0.0002 |
| rs17024393 | 1 | 110154688 | BDNF(B/M) | C/T | 0.03 | 0.93(0.87-0.98) | 0.007 |  | 0.41 | 0.96 (0.92-0.99) | 0.01 |  | 0.94(0.91-0.97) | 0.0003 |
| rs2867125 | 2 | 622827 | GNPDA2(N) | C/T | 0.83 | 0.96(0.94-0.99) | 0.003 |  | 0.168 | 0.97 (0.94-1.00) | 0.07 |  | 0.96(0.94-0.99) | 0.0008 |
| rs2287019 | 19 | 46202172 | LI NG02(D,N) | C/T | 0.79 | 0.96(0.93-0.99) | 0.009 |  | 0.80 | 0.96 (0.92-1.00) | 0.06 |  | 0.96(0.94-0.99) | 0.0010 |
| rs3810291 | 19 | 47569003 | CLIP1(N) | A/G | 0.67 | 0.98(0.95-1.00) | 0.01 |  | 0.43 | 0.96(0.92-0.99) | 0.01 |  | 0.97(0.95-0.99) | 0.002 |
| rs571312 | 18 | 57839769 | NT5C2(N) | A/C | 0.24 | 0.97(0.95-1.00) | 0.02 |  | 0.23 | 0.96 (0.92-1.00) | 0.04 |  | 0.97(0.95-0.99) | 0.002 |
| rs543874 | 1 | 177889480 | ELAVL4(B,D,N,Q) | G/A | 0.19 | 0.97(0.95-1.00) | 0.04 |  | 0.20 | 0.96 (0.92-1.00) | 0.04 |  | 0.97(0.95-0.99) | 0.005 |
| rs12401738 | 1 | 78446761 | HIP1(B,N) | A/G | 0.38 | 0.98(0.96-1.00) | 0.05 |  | 0.38 | 0.96(0.93-1.00) | 0.05 |  | 0.97(0.96-0.99) | 0.008 |
| rs1528435 | 2 | 181550962 | EHBP1(B,N) | T/C | 0.62 | 0.97(0.95-0.99) | 0.01 |  | 0.63 | 0.98(0.94-1.01) | 0.22 |  | 0.97(0.96-0.99) | 0.008 |
| rs2112347 | 5 | 75015242 | PRKDI(N) | T/G | 0.63 | 0.98(0.96-1.00) | 0.03 |  | 0.44 | 0.97 (0.94-1.00) | 0.08 |  | 0.98(0.96-0.99) | 0.008 |
| rs10733682 | 9 | 129460914 | FUBPI(N) | A/G | 0.49 | 0.97(0.95-0.99) | 0.009 |  | 0.47 | 0.99(0.95-1.02) | 0.41 |  | 0.98(0.96-0.99) | 0.01 |
| rs13191362 | 6 | 163033350 | GPRC5B(C/Q) | A/G | 0.88 | 1.03(1.00-1.06) | 0.047 |  | 0.87 | 1.04(0.98-1.09) | 0.18 |  | 1.03(1.01-1.06) | 0.02 |
| rs17405819 | 8 | 76806584 | PRKD1(N) | T/C | 0.69 | 0.97(0.95-1.00) | 0.02 |  | 0.69 | 0.99(0.95-1.02) | 0.5 |  | 0.98(0.96-1.00) | 0.02 |
| rs37316885 | 15 | 51748610 | CADM2 | A/G | 0.47 | 0.98(0.96-1.00) | 0.12 |  | 0.43 | 0.98 (0.94-1.01) | 0.15 |  | 0.98(0.96-1.00) | 0.04 |
| rs11057405 | 12 | 122781897 | SLC39A8(M,N,Q) | G/A | 0.90 | 1.02(0.99-1.06) | 0.21 |  | 0.90 | 1.05(0.99-1.11) | 0.1 |  | 1.03(1.00-1.06) | 0.05 |
| rs17001654 | 4 | 77129568 | PARK2(B,D,N) | G/C | 0.16 | 0.98(0.95-1.01) | 0.19 |  | 0.15 | 0.97(0.92-1.02) | 0.18 |  | 0.98(0.95-1.00) | 0.05 |
| rs657452 | 1 | 49589847 | MIR548A2(N) | A/G | 0.37 | 0.98(0.96-1.00) | 0.10 |  | 0.39 | 0.99(0.96-1.03) | 0.61 |  | 0.98(0.97-1.00) | 0.09 |
| rs11583200 | 1 | 50559820 | TNNI3K | C/T | 0.39 | 0.98(0.96-1.00) | 0.11 |  | 0.38 | 0.99(0.95-1.02) | 0.41 |  | 0.98(0.97-1.00) | 0.10 |
| rs1000940 | 17 | 5283252 | UBE2E3(N) | G/A | 0.30 | 1.01(0.99-1.03) | 0.30 |  | 0.30 | 1.03(0.99-1.07) | 0.11 |  | 1.02(1.00-1.03) | 0.10 |
| rs11191560 | 10 | 104869038 | PTBP2 | C/T | 0.09 | 0.98(0.94-1.01) | 0.20 |  | 0.53 | 0.99 (0.96-1.02) | 0.56 |  | 0.98(0.96-1.01) | 0.20 |
| rs16907751 | 8 | 81375457 | FTO(B,N) | C/T | 0.89 | 0.99(0.96-1.03) | 0.75 |  | 0.73 | 0.97(0.94-1.01) | 0.17 |  | 0.98(0.96-1.01) | 0.22 |
| rs10938397 | 4 | 45182527 | RASA2(N) | G/A | 0.43 | 0.99(0.97-1.01) | 0.47 |  | 0.83 | 0.96 (0.92-1.01) | 0.08 |  | 0.98(0.95-1.01) | 0.23 |
| rs29941 | 19 | 34309532 | ZBTB10(N) | G/A | 0.68 | 0.99(0.97-1.01) | 0.54 |  | 0.69 | 0.97(0.93-1.01) | 0.13 |  | 0.99(0.97-1.01) | 0.23 |
| rs4771122 | 13 | 28020180 | NUP54(M) | G/A | 0.25 | 0.98(0.96-1.00) | 0.06 |  | 0.03 | 0.85(0.75-0.96) | 0.003 |  | 0.92(0.79-1.06) | 0.23 |
| rs13078807 | 3 | 85884150 | GNAT2(N) | G/A | 0.19 | 0.99(0.97-1.02) | 0.67 |  | 0.19 | 0.98 (0.93-1.02) | 0.24 |  | 0.99(0.96-1.01) | 0.24 |
| rs2365389 | 3 | 612316862 | HIF1AN(N) | C/T | 0.58 | 0.99(0.97-1.01) | 0.53 |  | 0.68 | 0.97 (0.94-1.01) | 0.11 |  | 0.99(0.97-1.01) | 0.24 |
| rs7138803 | 12 | 50247468 | CREB1(B,N) | A/G | 0.39 | 0.99(0.97-1.01) | 0.19 |  | 0.38 | 1.00 (0.97-1.04) | 0.93 |  | 0.99(0.97-1.01) | 0.24 |
| rs1167827 | 7 | 75163169 | HNF4G(B,N) | G/A | 0.57 | 1.01(0.99-1.03) | 0.30 |  | 0.58 | 1.01(0.97-1.04) | 0.75 |  | 1.01(0.99-1.03) | 0.25 |
| rs11847697 | 14 | 30515112 | GDF15(B) | T/C | 0.06 | 0.96(0.90-1.03) | 0.24 |  | 0.04 | 0.99 (0.90-1.09) | 0.84 |  | 0.97(0.92-1.02) | 0.27 |
| rs2075650 | 19 | 45395619 | NPC1(B,G,M,Q) | A/G | 0.85 | 1.02(0.99-1.05) | 0.23 |  | 0.85 | 1.00(0.95-1.05) | 0.97 |  | 1.01(0.99-1.04) | 0.29 |
| rs17203016 | 2 | 208255518 | TLR4(B,N) | G/A | 0.20 | 1.02(0.99-1.04) | 0.24 |  | 0.20 | 1.00(0.95-1.04) | 0.91 |  | 1.01(0.99-1.03) | 0.32 |
| rs9816226 | 3 | 185834499 | C6orf106(N) | T/A | 0.82 | 1.01(0.98-1.04) | 0.41 |  | 0.83 | 1.01 (0.97-1.06) | 0.53 |  | 1.01(0.99-1.03) | 0.33 |
| rs10132280 | 14 | 25928179 | NUDT3 | C/A | 0.69 | 1.00(0.98-1.03) | 0.75 |  | 0.60 | 1.03(0.99-1.06) | 0.15 |  | 1.01(0.99-1.04) | 0.34 |
| rs99259168 | 16 | 31129895 | TOMM40(B,N) | A/G | 0.63 | 1.00(0.98-1.02) | 0.91 |  | 0.25 | 0.93 (0.89-0.97) | 0.002 |  | 0.97(0.90-1.04) | 0.36 |
| rs91681123 | 7 | 93197732 | CBLN1(N) | C/G | 0.41 | 1.00(0.98-1.02) | 0.96 |  | 0.43 | 0.95(0.92-0.99) | 0.01 |  | 0.98(0.93-1.03) | 0.37 |
| rs2176598 | 11 | 438168278 | P005(M) | T/C | 0.25 | 1.00(0.97-1.02) | 0.68 |  | 0.25 | 0.98(0.94-1.02) | 0.23 |  | 0.99(0.97-1.01) | 0.40 |
| rs12885454 | 14 | 29736838 | LOC1686736(N) | C/A | 0.168 | 1.01(0.99-1.03) | 0.50 |  | 0.17 | 1.01(0.98-1.05) | 0.47 |  | 1.01(0.99-1.03) | 0.41 |
| rs71168727 | 15 | 73093991 | HSD17B12(B,M,N) | T/C | 0.68 | 0.99(0.97-1.01) | 0.51 |  | 0.67 | 0.99(0.95-1.03) | 0.6 |  | 0.99(0.97-1.01) | 0.41 |
| rs13107325 | 4 | 103188709 | MAP2K5 | T/C | 0.07 | 1.00(0.96-1.04) | 0.93 |  | 0.48 | 0.98(0.94-1.01) | 0.17 |  | 0.99(0.96-1.02) | 0.42 |
| rs2836754 | 21 | 40291740 | QPCTL(N) | C/T | 0.168 | 1.00(0.98-1.02) | 0.71 |  | 0.41 | 1.02 (0.99-1.06) | 0.19 |  | 1.01(0.99-1.02) | 0.44 |
| rs7359397 | 16 | 28885659 | FHIT(N) | T/C | 0.39 | 1.01(0.99-1.03) | 0.55 |  | 0.37 | 1.01 (0.98-1.05) | 0.52 |  | 1.01(0.99-1.02) | 0.45 |
| rs14412168 | 13 | 79580919 | NEGR1(C/Q) | A/G | 0.17 | 0.99(0.96-1.01) | 0.39 |  | 0.60 | 1.00(0.96-1.03) | 0.95 |  | 0.99(0.97-1.01) | 0.47 |
| rs4836133 | 5 | 124332103 | NAV1(N) | A/C | 0.52 | 0.99(0.98-1.01) | 0.59 |  | 0.08 | 0.98(0.92-1.04) | 0.56 |  | 0.99(0.98-1.01) | 0.47 |
| rs16877694 | 9 | 111932342 | ETS2(N) | C/T | 0.36 | 1.00(0.98-1.02) | 0.79 |  | 0.35 | 0.98(0.95-1.02) | 0.4 |  | 0.99(0.98-1.01) | 0.49 |
| rs205262 | 6 | 345631168 | TMEM18 | G/A | 0.27 | 1.01(0.99-1.03) | 0.31 |  | 0.27 | 0.99(0.96-1.03) | 0.75 |  | 1.01(0.99-1.03) | 0.51 |
| rs7243357 | 18 | 56883319 | LRP1B | T/G | 0.83 | 1.00(0.97-1.02) | 0.92 |  | 0.83 | 0.98(0.94-1.03) | 0.48 |  | 0.99(0.97-1.01) | 0.53 |
| rs1928295 | 9 | 120378483 | KCTD15(N) | T/C | 0.55 | 1.00(0.98-1.02) | 0.85 |  | 0.57 | 0.98(0.95-1.02) | 0.32 |  | 0.99(0.98-1.01) | 0.53 |
| rs4740619 | 9 | 15634326 | SCG3(B,D) | T/C | 0.54 | 1.01(0.99-1.03) | 0.50 |  | 0.56 | 1.00(0.96-1.03) | 0.86 |  | 1.01(0.99-1.02) | 0.54 |
| rs3849570 | 3 | 81792112 | ZC3H4(D,N,Q) | A/C | 0.33 | 1.00(0.98-1.03) | 0.78 |  | 0.20 | 0.97 (0.93-1.02) | 0.27 |  | 0.99(0.96-1.02) | 0.56 |
| rs2176040 | 2 | 227092802 | MTCH2(M,Q) | A/G | 0.36 | 1.00(0.98-1.02) | 0.92 |  | 0.35 | 0.98(0.95-1.02) | 0.37 |  | 1.00(0.98-1.01) | 0.59 |
| rs1514175 | 1 | 749911684 | GBE1(B,M,N) | A/G | 0.42 | 1.00(0.98-1.02) | 1.00 |  | 0.07 | 0.95 (0.88-1.02) | 0.16 |  | 0.99(0.95-1.03) | 0.62 |
| rs887912 | 2 | 59302877 | C9orf93(C,M,N) | T/C | 0.29 | 1.00(0.98-1.02) | 0.78 |  | 0.30 | 0.99 (0.96-1.03) | 0.66 |  | 1.00(0.98-1.01) | 0.63 |
| rs12444979 | 16 | 19933600 | MTIF3 | C/T | 0.86 | 0.99(0.96-1.02) | 0.49 |  | 0.86 | 1.01 (0.96-1.06) | 0.62 |  | 0.99(0.97-1.02) | 0.63 |
| rs107676168 | 11 | 27725986 | MAPK3(D) | A/T | 0.78 | 0.99(0.97-1.02) | 0.54 |  | 0.79 | 1.04 (0.99-1.08) | 0.11 |  | 1.01(0.97-1.04) | 0.67 |
| rs2815752 | 1 | 72812440 | ZNF608 | A/G | 0.61 | 0.99(0.97-1.01) | 0.22 |  | 0.18 | 1.04 (1.00-1.09) | 0.06 |  | 1.01(0.96-1.06) | 0.67 |
| rs987237 | 6 | 50803050 | PLCD4(B,Q) | G/A | 0.18 | 1.01(0.98-1.03) | 0.52 |  | 0.62 | 0.97 (0.93-1.00) | 0.06 |  | 0.99(0.96-1.03) | 0.68 |
| rs11688816 | 2 | 63053048 | RPL27A | G/A | 0.53 | 1.01(0.99-1.03) | 0.49 |  | 0.54 | 0.99(0.96-1.03) | 0.74 |  | 1.00(0.99-1.02) | 0.69 |
| rs1808579 | 18 | 21104888 | SEC16B(N) | C/T | 0.53 | 1.01(0.99-1.03) | 0.40 |  | 0.53 | 0.98(0.94-1.01) | 0.15 |  | 0.99(0.96-1.03) | 0.72 |
| rs492400 | 2 | 219349752 | MC4R(B) | C/T | 0.41 | 1.02(0.99-1.04) | 0.15 |  | 0.17 | 0.96(0.92-0.99) | 0.02 |  | 0.99(0.94-1.04) | 0.72 |
| rs4787491 | 16 | 30015337 | EPB41L4B(N) | G/A | 0.54 | 1.00(0.98-1.02) | 0.90 |  | 0.54 | 1.02(0.98-1.06) | 0.35 |  | 1.00(0.99-1.02) | 0.73 |
| rs2890652 | 2 | 142959931 | AGBL4(N) | C/T | 0.17 | 1.00(0.97-1.02) | 0.77 |  | 0.17 | 1.02 (0.97-1.06) | 0.48 |  | 1.00(0.98-1.03) | 0.74 |
| rs9374842 | 6 | 120185665 | RBJ | T/C | 0.77 | 1.01(0.99-1.04) | 0.27 |  | 0.78 | 0.97(0.93-1.01) | 0.17 |  | 0.99(0.95-1.04) | 0.80 |
| rs977747 | 1 | 47684677 | BCDIN3D(N) | T/G | 0.39 | 1.00(0.98-1.02) | 0.81 |  | 0.42 | 0.98(0.95-1.02) | 0.35 |  | 1.00(0.98-1.02) | 0.81 |
| rs1555543 | 1 | 96944797 | LOC100287559(N) | C/A | 0.60 | 1.00(0.97-1.02) | 0.81 |  | 0.58 | 1.00 (0.97-1.04) | 0.97 |  | 1.00(0.98-1.02) | 0.83 |
| rs2241423 | 15 | 68086838 | LOC284260(N) | G/A | 0.77 | 1.00(0.98-1.03) | 0.69 |  | 0.78 | 0.98 (0.94-1.02) | 0.23 |  | 1.00(0.98-1.02) | 0.83 |
| rs758747 | 16 | 3627358 | GRP(B,G,N) | T/C | 0.27 | 1.00(0.97-1.02) | 0.68 |  | 0.27 | 1.02(0.98-1.06) | 0.37 |  | 1.00(0.98-1.02) | 0.85 |
| rs9914578 | 17 | 2005136 | SH2B1(Q/B/M) | G/C | 0.20 | 1.02(1.00-1.04) | 0.09 |  | 0.20 | 0.99(0.94-1.03) | 0.48 |  | 1.00(0.96-1.04) | 0.87 |
| rs17094222 | 10 | 102395440 | NLRC3(N) | C/T | 0.22 | 1.01(0.98-1.04) | 0.54 |  | 0.21 | 0.98(0.94-1.02) | 0.39 |  | 1.00(0.97-1.02) | 0.88 |
| rs7899106 | 10 | 87410904 | ERBB4(D,N) | G/A | 0.05 | 1.00(0.95-1.05) | 0.99 |  | 0.05 | 1.01(0.93-1.09) | 0.87 |  | 1.00(0.96-1.04) | 0.89 |
| rs10150332 | 14 | 799369168 | GALNT10(N) | C/T | 0.21 | 1.02(1.00-1.04) | 0.12 |  | 0.21 | 0.98 (0.94-1.02) | 0.35 |  | 1.00(0.97-1.04) | 0.89 |
| rs9400239 | 6 | 108977663 | GRID1(B,N) | C/T | 0.70 | 1.00(0.97-1.02) | 0.67 |  | 0.71 | 1.01(0.97-1.05) | 0.168 |  | 1.00(0.98-1.02) | 0.89 |
| rs17724992 | 19 | 18454825 | TCF7L2(B,N) | A/G | 0.75 | 1.01(0.98-1.03) | 0.56 |  | 0.43 | 0.99(0.95-1.02) | 0.39 |  | 1.00(0.97-1.02) | 0.90 |
| rs7239883 | 18 | 40147671 | FANCL | G/A | 0.40 | 1.00(0.98-1.02) | 0.89 |  | 0.38 | 1.00(0.97-1.03) | 1 |  | 1.00(0.98-1.02) | 0.90 |
| rs4929949 | 11 | 8604593 | LOC285762(N) | C/T | 0.51 | 1.00(0.98-1.02) | 0.90 |  | 0.52 | 1.01 (0.97-1.04) | 0.69 |  | 1.00(0.98-1.02) | 0.91 |
| rs3817334 | 11 | 47650993 | FOXO3(B,N) | T/C | 0.41 | 1.01(0.98-1.03) | 0.51 |  | 0.168 | 0.98(0.94-1.01) | 0.21 |  | 1.00(0.97-1.02) | 0.92 |
| rs7715256 | 5 | 153537893 | CALCR(B,N) | G/T | 0.42 | 0.99(0.97-1.01) | 0.53 |  | 0.74 | 1.02(0.98-1.06) | 0.38 |  | 1.00(0.98-1.02) | 0.92 |
| rs16851483 | 3 | 141275436 | TAL1(N) | T/G | 0.06 | 1.03(0.99-1.07) | 0.15 |  | 0.07 | 0.96(0.89-1.03) | 0.3 |  | 1.00(0.94-1.07) | 0.93 |
| rs10968576 | 9 | 28414339 | ETV5 | G/A | 0.31 | 1.00(0.98-1.02) | 0.93 |  | 0.32 | 1.00 (0.96-1.04) | 0.94 |  | 1.00(0.98-1.02) | 0.94 |
| rs206936 | 6 | 34302869 | TFAP2B | G/A | 0.20 | 0.99(0.97-1.01) | 0.43 |  | 0.34 | 1.02(0.98-1.06) | 0.27 |  | 1.00(0.97-1.03) | 0.95 |
| rs2820292 | 1 | 201784287 | SMG6(D,N) | C/A | 0.56 | 1.00(0.98-1.02) | 0.85 |  | 0.55 | 1.01(0.97-1.04) | 0.77 |  | 1.00(0.98-1.02) | 0.95 |
| rs2080454 | 16 | 49062590 | KAT8(N) | C/A | 0.43 | 1.00(0.98-1.02) | 0.95 |  | 0.38 | 1.00(0.96-1.03) | 0.9 |  | 1.00(0.98-1.02) | 0.96 |
| rs713586 | 2 | 25158008 | STXBP6(N) | C/T | 0.47 | 0.94(0.92-0.97) | 1.82×10^-6^ |  | 0.48 | 0.96 (0.93-1.00) | 0.03 |  | 0.95(0.93-0.97) | 3.19×10^-7^ |
| rs1558902 | 16 | 53803574 | RABEP1(N) | A/T | 0.41 | 0.93(0.91-0.95) | 2.77×10^-14^ |  | 0.68 | 0.95 (0.91-0.99) | 0.01 |  | 0.93(0.91-0.95) | 3.63×10^-16^ |
| rs7903146 | 10 | 114758349 | NRXN3 | C/T | 0.72 | 0.96(0.94-0.98) | 7.01×10^-5^ |  | 0.70 | 0.96(0.92-1.00) | 0.04 |  | 0.96(0.94-0.98) | 8.65×10^-6^ |
| *Results are presented for per allele increase of SNP. | | | | | | | | | | | | | | |
