# Supplementary material for: Genetically Predicted Body Mass Index and Breast Cancer Risk: Mendelian Randomization Analyses of Data from 145,000 Women of European Descent
Source: PLoS Med. 2016 Aug 23;13(8):e1002105. doi: 10.1371/journal.pmed.1002105 (PMC4995025; doi:10.1371/journal.pmed.1002105)
Supplement: S11 Table — (DOCX) [file pmed.1002105.s012.docx]

| **S11 Table. The associations between observed BMI and breast cancer risk using BCAC data (pooled analysis).** | | | | | | | |
| --- | --- | --- | --- | --- | --- | --- | --- |
|  |  |  |  | **Observed BMI*** | | **Observed BMI†** | |
|  |  | **Cases** | **Controls** | **OR(95%CI)** | **p** | **OR(95%CI)** | **p** |
| Prospective Cohort Studies | Premenopausal | 333 | 295 | 0.93(0.78-1.10) | 0.38 | 0.92(0.77-1.10) | 0.37 |
|  | Postmenopausal | 763 | 690 | 1.16(1.04-1.30) | 0.01 | 1.17(1.04-1.31) | 0.01 |
|  | Never HT use | 453 | 402 | 1.19(1.03-1.37) | 0.02 | 1.18(1.02-1.36) | 0.03 |
|  | Ever HT use | 280 | 261 | 1.09(0.89-1.34) | 0.4 | 1.10(0.89-1.34) | 0.33 |
|  | No HT use data | 30 | 27 | 1.51(0.75-2.81) | 0.26 | 1.54(0.75-2.87) | 0.24 |
| Non-prospective Studies | Premenopausal | 7375 | 5228 | 0.90(0.86-0.94) | <0.0001 | 0.91(0.87-0.94) | <0.0001 |
|  | Postmenopausal | 15710 | 14317 | 0.99(0.96-1.01) | 0.26 | 0.99(0.97-1.02) | 0.46 |
|  | Never HT use | 5546 | 4327 | 0.96(0.92-0.99) | 0.03 | 0.96(0.92-0.99) | 0.04 |
|  | Ever HT use | 5424 | 4307 | 0.88(0.84-0.93) | <0.0001 | 0.89(0.85-0.93) | <0.0001 |
|  | No HT use data | 4740 | 5683 | 1.12(1.07-1.17) | <0.0001 | 1.12(1.07-1.17) | <0.0001 |
| Results are presented for per 5kg/m2 increase of observed BMI. OR: odds ratio; CI: confidence interval.*Model was adjusted for age, study PC1-PC8. **†** Model was additionally adjusted for genetically predicted BMI. | | | | | | | |
