# Supplementary material for: Genetically Predicted Body Mass Index and Breast Cancer Risk: Mendelian Randomization Analyses of Data from 145,000 Women of European Descent
Source: PLoS Med. 2016 Aug 23;13(8):e1002105. doi: 10.1371/journal.pmed.1002105 (PMC4995025; doi:10.1371/journal.pmed.1002105)
Supplement: S1 Text — (DOCX) [file pmed.1002105.s013.docx]

**Funding**:

The work conducted for this project at Vanderbilt Epidemiology Center is supported in part by National Cancer Institute at the National Institutes of Health (grant number R37CA070867) and endowment funds for the Ingram Professorship and Anne Potter Wilson Chair. YG was supported by Cancer Center Support Grant (P30 CA068485). SWA was supported by the Vanderbilt Molecular and Genetic Epidemiology of Cancer training program (R25 CA160056).This work was partly supported by the European Community's Seventh Framework Programme under grant agreement number 223175 (grant number HEALTH-F2-2009-223175) (COGS). Funding for the iCOGS infrastructure came from: the European Community's Seventh Framework Programme under grant agreement n° 223175 (HEALTH-F2-2009-223175) (COGS), Cancer Research UK (C1287/A10118, C1287/A 10710, C12292/A11174, C1281/A12014, C5047/A8384, C5047/A15007, C5047/A10692), the National Institutes of Health (CA128978) and Post-Cancer GWAS initiative (1U19 CA148537, 1U19 CA148065 and 1U19 CA148112 - the GAME-ON initiative), the Department of Defence (W81XWH-10-1-0341), the Canadian Institutes of Health Research (CIHR) for the CIHR Team in Familial Risks of Breast Cancer, Komen Foundation for the Cure, the Breast Cancer Research Foundation, and the Ovarian Cancer Research Fund.

The **GAME-ON DRIVE** Consortium was supported by National Cancer Institute at the National Institutes of Health (grant number U19 CA148065). The work of the **BCFR** included in the DRIVE Project was supported by National Cancer Institute at the National Institutes of Health (grant UM1 CA164920)(USA). The content of this manuscript does not necessarily reflect the views or policies of the National Cancer Institute or any of the collaborating centers in the Breast Cancer Family Registry (BCFR), nor does mention of trade names, commercial products, or organizations imply endorsement by the USA Government or the BCFR. The **ABCFS**, **NC-BCFR** and **OFBCR** work was supported by National Cancer Institute at the National Institutes of Health (grant UM1 CA164920). The content of this manuscript does not necessarily reflect the views or policies of the National Cancer Institute or any of the collaborating centers in the Breast Cancer Family Registry (BCFR), nor does mention of trade names, commercial products, or organizations imply endorsement by the US Government or the BCFR. The ABCFS was also supported by the National Health and Medical Research Council of Australia, the New South Wales Cancer Council, the Victorian Health Promotion Foundation (Australia) and the Victorian Breast Cancer Research Consortium. JLH is a National Health and Medical Research Council (NHMRC) Australia Fellow and a Victorian Breast Cancer Research Consortium Group Leader. MCS is a NHMRC Senior Research Fellow and a Victorian Breast Cancer Research Consortium Group Leader. The **ABCS** study was supported by the Dutch Cancer Society [grants NKI 2007-3839; 2009 4363]; BBMRI-NL, which is a Research Infrastructure financed by the Dutch government (NWO 184.021.007); and the Dutch National Genomics Initiative. The work of the **BBCC** was partly funded by ELAN-Fond of the University Hospital of Erlangen. The **BBCS** is funded by Cancer Research UK and Breakthrough Breast Cancer and acknowledges NHS funding to the NIHR Biomedical Research Centre, and the National Cancer Research Network (NCRN). The **BCAC** is funded by CR-UK (C1287/A10118 and C1287/A12014). Meetings of the BCAC have been funded by the European Union COST programme (BM0606). DFE is a Principal Research Fellow of CR-UK. In the **BIGGS**, ES is supported by NIHR Comprehensive Biomedical Research Centre, Guy's & St. Thomas' NHS Foundation Trust in partnership with King's College London, United Kingdom. IT is supported by the Oxford Biomedical Research Centre. The **BSUCH** study was supported by the Dietmar-Hopp Foundation, the Helmholtz Society and the German Cancer Research Center (DKFZ). The **CECILE** study was funded by Fondation de France, Institut National du Cancer (INCa), Ligue Nationale contre le Cancer, Ligue contre le Cancer Grand Ouest, Agence Nationale de Sécurité Sanitaire (ANSES), Agence Nationale de la Recherche (ANR). The **CGPS** was supported by the Chief Physician Johan Boserup and Lise Boserup Fund, the Danish Medical Research Council and Herlev Hospital. The **CNIO-BCS** was supported by the Genome Spain Foundation, the Red Temática de Investigación Cooperativa en Cáncer and grants from the Asociación Española Contra el Cáncer and the Fondo de Investigación Sanitario (PI11/00923 and PI081120). The Human Genotyping-CEGEN Unit (CNIO) is supported by the Instituto de Salud Carlos III. The **CTS** was initially supported by the California Breast Cancer Act of 1993 and the California Breast Cancer Research Fund (contract 97-10500) and is currently funded through the National Institutes of Health (R01 CA77398). Collection of cancer incidence data was supported by the California Department of Public Health as part of the statewide cancer reporting program mandated by California Health and Safety Code Section 103885. HAC receives support from the Lon V Smith Foundation (LVS39420). The **ESTHER** study was supported by a grant from the Baden Württemberg Ministry of Science, Research and Arts. Additional cases were recruited in the context of the VERDI study, which was supported by a grant from the German Cancer Aid (Deutsche Krebshilfe). The **GC-HBOC** was supported by Deutsche Krebshilfe (107 352). The **GENICA** was funded by the Federal Ministry of Education and Research (BMBF) Germany grants 01KW9975/5, 01KW9976/8, 01KW9977/0 and 01KW0114, the Robert Bosch Foundation, Stuttgart, Deutsches Krebsforschungszentrum (DKFZ), Heidelberg, Institute for Prevention and Occupational Medicine of the German Social Accident Insurance, Institute of the Ruhr University Bochum (IPA), Bochum, as well as the Department of Internal Medicine, Evangelische Kliniken Bonn gGmbH, Johanniter Krankenhaus, Bonn, Germany. The **HEBCS** was financially supported by the Helsinki University Central Hospital Research Fund, Academy of Finland (266528), the Finnish Cancer Society, The Nordic Cancer Union and the Sigrid Juselius Foundation. The **HMBCS** was supported by a grant from the Friends of Hannover Medical School and by the Rudolf Bartling Foundation. Financial support for **KARBAC** was provided through the regional agreement on medical training and clinical research (ALF) between Stockholm County Council and Karolinska Institutet, the Swedish Cancer Society, The Gustav V Jubilee foundation and and Bert von Kantzows foundation. The **KBCP** was financially supported by the special Government Funding (EVO) of Kuopio University Hospital grants, Cancer Fund of North Savo, the Finnish Cancer Organizations, and by the strategic funding of the University of Eastern Finland. The **kConFab** is supported by a grant from the National Breast Cancer Foundation, and previously by the National Health and Medical Research Council (NHMRC), the Queensland Cancer Fund, the Cancer Councils of New South Wales, Victoria, Tasmania and South Australia, and the Cancer Foundation of Western Australia. The kConFab Clinical Follow Up Study was funded by the NHMRC [145684, 288704, 454508]. Financial support for the **AOCS** was provided by the United States Army Medical Research and Materiel Command [DAMD17-01-1-0729], the Cancer Council of Tasmania and Cancer Foundation of Western Australia and the NHMRC [199600]. GCT and PW are supported by the NHMRC. **LMBC** is supported by the 'Stichting tegen Kanker' (232-2008 and 196-2010). Diether Lambrechts is supported by the FWO and the KULPFV/10/016-SymBioSysII. The **MARIE** study was supported by the Deutsche Krebshilfe e.V. [70-2892-BR I , 106332, 108253, 108419], the Hamburg Cancer Society, the German Cancer Research Center (DKFZ), and the Federal Ministry of Education and Research (BMBF) Germany [01KH0402]. **MBCSG** is supported by grants from the Italian Association for Cancer Research (AIRC) and by funds from the Italian citizens who allocated the 5/1000 share of their tax payment in support of the Fondazione IRCCS Istituto Nazionale Tumori, according to Italian laws (INT-Institutional strategic projects “5x1000”). **MBCSG** is supported by grants from the Italian Association for Cancer Research (AIRC). **MCBCS** was supported by the NIH grants CA116167, CA176785, CA192393 an NIH Specialized Program of Research Excellence (SPORE) in Breast Cancer [CA116201], the Breast Cancer Research Foundation and a generous gift from the David F. and Margaret T. Grohne Family Foundation. **MCCS** cohort recruitment in the study was funded by VicHealth and Cancer Council Victoria. The MCCS was further supported by Australian NHMRC grants 209057, 251553 and 504711 and by infrastructure provided by Cancer Council Victoria. The **MEC** was support by NIH grants CA63464, CA54281, CA098758 and CA132839. The work of **MTLGEBCS** was supported by the Quebec Breast Cancer Foundation, the Canadian Institutes of Health Research for the “CIHR Team in Familial Risks of Breast Cancer” program – grant # CRN-87521 and the Ministry of Economic Development, Innovation and Export Trade – grant # PSR-SIIRI-701. The **NBCS** was supported by grants from the Norwegian Research Council (grant numbers 183621/S10 and 175240/S10), the Norwegian Cancer Society (grant numbers 419616 and 419628) and the KG Jebsen Center for Breast Cancer Research. The NBHS was supported by NIH grant R01CA100374. Biological sample preparation was conducted the Survey and Biospecimen Shared Resource, which is supported by P30 CA68485.

The **OBCS** was supported by research grants from the Finnish Cancer Foundation, the Academy of Finland, the University of Oulu, and the Oulu University Hospital. The **OFBCR** was supported by grant UM1 CA164920 from the National Cancer Institute. The content of this manuscript does not necessarily reflect the views or policies of the National Cancer Institute or any of the collaborating centers in the Breast Cancer Family Registry (BCFR), nor does mention of trade names, commercial products, or organizations imply endorsement by the US Government or the BCFR. The **ORIGO** study was supported by the Dutch Cancer Society (RUL 1997-1505) and the Biobanking and Biomolecular Resources Research Infrastructure (BBMRI-NL CP16). The **PBCS** was funded by Intramural Research Funds of the National Cancer Institute, Department of Health and Human Services, USA. The **pKARMA** study was supported by Märit and Hans Rausings Initiative Against Breast Cancer. The **RBCS** was funded by the Dutch Cancer Society (DDHK 2004-3124, DDHK 2009-4318). The **SASBAC** study was supported by funding from the Agency for Science, Technology and Research of Singapore (A*STAR), the US National Institute of Health (NIH) and the Susan G. Komen Breast Cancer Foundation. The **SBCS** was supported by Yorkshire Cancer Research S295, S299, and S305PA and by the Sheffield Experimental Cancer Medicine Centre. **SEARCH** is funded by a programme grant from Cancer Research UK [C490/A10124] and supported by the UK National Institute for Health Research Biomedical Research Centre at the University of Cambridge. **SKKDKFZS** is supported by the DKFZ. The **SZBCS** was supported by Grant PBZ_KBN_122/P05/2004. The **TNBCC** was supported by: a Specialized Program of Research Excellence (SPORE) in Breast Cancer (CA116201), a grant from the Breast Cancer Research Foundation, a generous gift from the David F. and Margaret T. Grohne Family Foundation, the Stefanie Spielman Breast Cancer fund and the OSU Comprehensive Cancer Center, the Hellenic Cooperative Oncology Group research grant (HR R_BG/04) and the Greek General Secretary for Research and Technology (GSRT) Program, Research Excellence II, the European Union (European Social Fund – ESF), and Greek national funds through the Operational Program "Education and Lifelong Learning" of the National Strategic Reference Framework (NSRF) - ARISTEIA.The **UKBGS** is funded by Breakthrough Breast Cancer and the Institute of Cancer Research (ICR). ICR acknowledges NHS funding to the NIHR Biomedical Research Centre. The **DFBBCS** GWAS was funded by The Netherlands Organisation for Scientific Research (NWO) as part of a ZonMw/VIDI grant number 91756341. The generation and management of GWAS genotype data for the Rotterdam Study (control samples) is supported by the Netherlands Organisation of Scientific Research NWO Investments (nr. 175.010.2005.011, 911-03-012). This study is funded by the Research Institute for Diseases in the Elderly (014-93-015; RIDE2), the Netherlands Genomics Initiative (NGI)/Netherlands Organisation for Scientific Research (NWO) project nr. 050-060-810.
